# Integration of a brief, transdiagnostic psychological intervention in the care of adolescents and young adults with HIV in Kenya: Protocol for a cluster randomized clinical trial

**DOI:** 10.1371/journal.pone.0325374

**Published:** 2025-06-20

**Authors:** Cyrus Mugo, Irene Njuguna, Vincent Maganga, Brian P. Flaherty, Anjuli D. Wagner, Ferdinand C. Mukumbang, Shannon Dorsey, Daniel Mwai, Caren Mburu, Manasi Kumar, Anne Mbwayo, Muthoni Mathai, Pamela Y. Collins, Dalton Wamalwa

**Affiliations:** 1 Department of Medical Research, Kenyatta National Hospital, Nairobi, Kenya; 2 Department of Global Health, University of Washington, Seattle, Washington, United States of America; 3 Department of Pediatrics and Child Health, University of Nairobi, Nairobi, Kenya; 4 Department of Psychology, University of Washington, Seattle, Washington, United States of America; 5 School of Global Health, University of Nairobi, Nairobi, Kenya; 6 Elizabeth Glaser Pediatric AIDS Foundation, Nairobi, Kenya; 7 Institute for Excellence in Health Equity, New York University, New York, United States of America; 8 Department of Psychiatry and Mental Health, University of Nairobi, Nairobi, Kenya; 9 Department of Mental Health, Johns Hopkins University, Maryland, United States of America; PLOS: Public Library of Science, UNITED KINGDOM OF GREAT BRITAIN AND NORTHERN IRELAND

## Abstract

Adolescents and young adults with HIV (AYH) are at greater risk for mental health conditions. Provision of mental health services to AYH is limited by an overburdened and untrained workforce, and psychological interventions ill-adapted for adolescent HIV care. Brief, transdiagnostic psychological interventions delivered in the HIV clinics may address some of these challenges. We will evaluate the PRO-ACT intervention in a hybrid type 1 cluster randomized clinical trial (NCT06247527) in 30 HIV clinics in 3 counties in Kenya. PRO-ACT is a cognitive behavioral therapy with 5 modules: Psychoeducation, Relaxation, prOblem-solving, behavioral Activation and Cognitive coping, delivered to AYH by trained health providers without specialist mental health training in 4–6 sessions within 6 months. We performed stratified randomization with matching to balance for county and facility size to assign 5 intervention (PRO-ACT) and 5 control (standard of care) clinics in each of the 3 counties. Screening of AYH ages 16−24 years will be conducted using the 9-item patient health questionnaire (PHQ-9). Up to 300 AYH with psychological distress (PHQ-9 score>4) will be enrolled in each arm. Further assessments will include the 5-item ASK suicide screening questions (ASQ), 7-item Generalized Anxiety Disorder (GAD-7), 20-item Child and Adolescent Trauma Screen (CATS)/PTSD Checklist for DSM-5 (PCL-5), self-reported treatment adherence, and HIV viral loads. Follow-up assessments will be conducted at month 3, 6, 9 and 12. Mixed methods will be used to measure implementation outcomes, cost-effectiveness, and characterize determinants of implementation. Primary analysis of PRO-ACT effectiveness will be conducted at month 6 (near term treatment effect), and month 12 (treatment effect sustainability) comparing PHQ-9 mean scores between treatment and control groups. Secondary analyses will compare GAD-7, CATS/PCL-5, adherence and viral suppression between the groups. The study will inform integration efforts of holistic mental health services in HIV care.

## Introduction

Mental disorders are a major cause of morbidity among adolescents (age 10–19) and young adults (age 20-24 years) with HIV (AYH) [1–3], the majority of whom reside in sub-Saharan Africa (SSA) [4]. Recent studies report prevalence of mental health conditions – including depression, trauma and anxiety – among AYH to be over 2-fold higher than among young adults not living with HIV, ranging from 25–50% [1,2,5–7]. There is strong evidence that mental health conditions are linked to poor HIV clinical outcomes, including treatment adherence, viral suppression and mortality, hence the urgent need to address mental health as part of routine clinical care for AYH [8–13].

Adolescents and young adults experience intersecting vulnerability to HIV and co-occurring mental health conditions. This vulnerability is potentially linked to rapid neuropsychological and social development during which young adults seek greater autonomy and are more likely to initiate sexual activity [9,14]. Adolescents and young adults ages 15–24 years contributed to 3 of every 7 new HIV infections globally, the highest incidence compared to other age groups in 2023 [4]. AYH who were perinatally infected often learn about their HIV diagnosis in adolescence, which, if unsupported, is associated with coping challenges [15,16]. Additionally, AYH are expected to transition to care in adult HIV clinics, which are often unwelcoming and stigmatizing [15–17]. Other major life transitions such as beginning high school or boarding school and initiating romantic relationships could further compound mental health challenges among AYH [18,19]. Other risk factors for poor mental health among AYH include coping with an HIV diagnosis, discrimination due to HIV-related stigma, biological and social effects of the disease progression, exposure to violence, poverty, poor quality of parenting, substance use and caregiver’s poor mental health [3,20–26].

Effective mental health treatments, including psychotherapy, exist and have been linked to improvement in ART adherence among AYH [27–29]. However, as in the majority of settings in SSA, routine screening and management of mental disorders is limited to a few well-resourced tertiary centers with few trained mental health personnel [30]. The inflexible cadence of clinic visits for school-going AYH [19] and high workload in HIV clinics [31]. limit the opportunities for mental health screening for AYH. Low- and Middle-Income Countries (LMICs), like Kenya, are characterized by a wide mental health treatment gap, and subsequently there is a dearth of trained mental health personnel to provide mental health care to AYH. This necessitates the need for task shifting and briefer interventions that are flexible enough to address multiple and/or comorbid mental health problems. Pragmatic interventions that fit HIV clinic needs and settings are necessary to address the mental health treatment gap for AYH.

Transdiagnostic interventions, which address multiple mental health conditions, may be preferable to diagnostic-specific interventions, as AYH rarely present with only one mental health problem; rather, they more likely present with co-occurring depressive, anxiety and/or trauma symptoms [32]. Thus, single-disorder-focused treatments may not match the more common clinical need in which mental health conditions overlap. Furthermore, many interventions have a ‘cross-over effect’, where targeting depression also reduces anxiety and trauma related symptoms and vice versa [33,34]. Studies among adolescents and young adults in high-income countries have found brief psychological interventions effective in treatment of emotional disorders [35], while some in low-income settings have shown promise [36–38]. As part of the World Health Organization’s ‘Ensuring Quality in Psychological Support’ (WHO EQUIP) project [39], a pilot study tested the feasibility of community health volunteers being trained in and delivering a brief transdiagnostic intervention PRO-ACT for Kenyan adolescents and young adults with mild to moderate psychological distress. The brief Cognitive Behavioral Therapy (CBT) based intervention, PRO-ACT, showed promise in reducing depressive, anxiety, and trauma symptoms. Findings from this pilot provide a foundation for larger effectiveness trials of transdiagnostic interventions. In this paper, we describe the study protocol for a cluster randomized trial testing the effectiveness of PRO-ACT in reducing mental health symptoms among AYH, when delivered by formally trained HIV clinic staff and including peers. We describe the implementation and expected client outcomes that will be measured along with cost-effectiveness in a population of Kenyan AYH.

## Methods

### Study design

We will conduct a hybrid implementation-effectiveness type 1 cluster randomized clinical trial (cRCT) [40] to evaluate the effectiveness, implementation, cost and cost-effectiveness of the PRO-ACT intervention. The intervention is intended to reduce depressive, anxiety, and trauma-related symptoms among AYH in selected HIV clinics in Nairobi, Kisumu and Homa Bay counties in Kenya. The intervention will be compared to standard of care.

**Fig 1:**
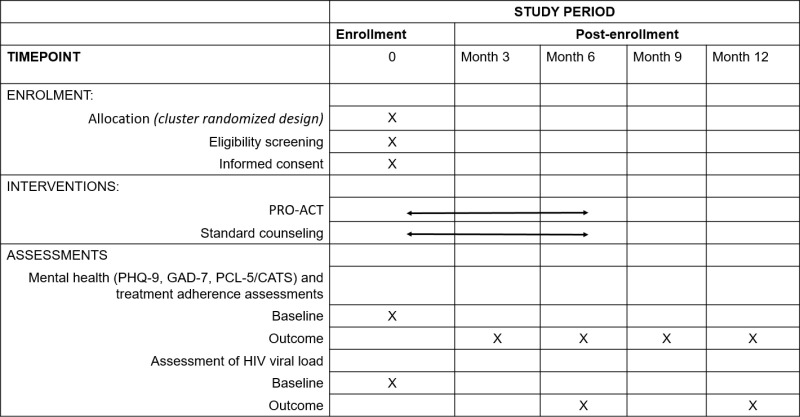
PRO-ACT schedule of enrolment, interventions and assessments.

The trial is registered at ClinicalTrials.gov (NCT06247527). The study was approved by the Kenyatta National Hospital-University of Nairobi Ethics and Research Committee (KNH-UoN ERC) (P509/05/2023) and the University of Washington Institutional Review Board (STUDY00018257); protocol version 4, January 7, 2025. Approval was also received from the National Commission on Science and Technology (NACOSTI), County Health Departments, and facility heads. The trial is overseen by a data safety and monitoring board (DSMB) constituted by the funder – National Institute of Mental Health, and composed of mental health researchers and a biostatistician. Recruitment and enrollment of participants to the trial started in August 2024 and is expected to be completed by December 2025, with follow up of participants (data collection) expected to end by December 2026, and results dissemination by April 2027. The study’s schedule of enrolment, interventions and assessments is summarized in Fig 1.

### The intervention

PRO-ACT is a brief trans-diagnostic psychological intervention, with the included elements overlapping those in the “stabilization phase” of trauma-focused cognitive behavioral therapy (TF-CBT), with TF-CBT having demonstrated effectiveness in studies in the region [41], but more complex and longer in duration than desirable for mental health care in HIV clinics. The PRO-ACT intervention is delivered in-person (preferred) or via telephone in 4–6 sessions, over 6 or fewer months by a non-specialist provider trained and offered support supervision.

The modules include 1) Psychoeducation, which begins the provider-client relationship, empowers the client with information on diagnosis, symptoms and knowledge of etiology, while the client receives normalization and validation of feelings, and hope; 2) Relaxation, where AYH learn to recognize how stress is experienced in the body, learn to recognize and rate severity of stress/body tension, and learn practical ways to relax the body; 3) Problem solving, where the counselor uses a problem-solving framework to help young adults identify and define problems causing anxiety and depression, break them down to smaller sub-problems, taking perspective of others, and brainstorm possible solutions to the problem to enact/try; 4) Behavioral activation, where the counselor supports young adults to increase engagement in social/other activities that bring them meaning or pleasure, identify activities that the young adults can integrate in their day-to-day program and rating the impact of various activities on their mood; 5) Cognitive coping, where AYH learn that they can change their thinking to feel better; Learn how thoughts, feelings, and behavior are connected; Learn how changing thought can change the way one feels and consequently the way one behaves. These active components (psychoeducation, relaxation, problem-solving, behavioral activation and coping) share the mechanisms of action of other CBT-based therapies that reduce anxiety, depressive symptoms, and trauma-related anxiety symptom [38,41].

**Fig 2:**
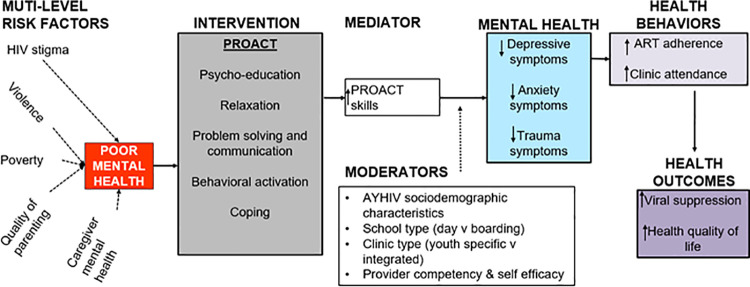
PRO-ACT conceptual framework.

We hypothesize that AYH experiencing distress linked to multiple risk factors, including HIV-related vulnerabilities like HIV stigma and discrimination, will gain PRO-ACT skills with practice. These new behaviors and thinking styles will reduce anxiety and depressive symptoms and increase the likelihood of improved adherence and retention in care, which will in turn improve viral suppression and quality of life. Fig 2 describes our conceptual framework.

### Standard of care practices

The HIV care guidelines [42,43] require that AYH are screened every 6 months (during routine study visits) for depression using the 9-item patient health questionnaire (PHQ-9). Although the PHQ-9 has been integrated for routine screening in the HIV clinics, this is hardly or very erratically implemented. The guidelines require that AYH with severe depression (PHQ-9 score >19) when detected are referred either to a mental health provider within the facility or outside the facility. Majority of mental health providers are adherence counselors with limited skills to address depression and other common mental disorders. When accessible, the providers offer different types of interventions, including problem solving and supportive counseling, based on their preference and training. Additionally, access to mental health providers varies depending on availability of the provider in the HIV clinic, facility size, and the distance to the referral facility. AYH with poor treatment adherence and high viral loads are frequently referred to the counselors within the facilities whether or not they are screened for depression.

### Site selection and randomization

Study sites were selected in Nairobi City, Kisumu City, and Homa Bay Counties in Kenya, which are among the top five counties in Kenya with the highest numbers of AYH in care [44]. Each county health department provided the study with aggregated monthly data for each facility on the total number of patients, ages 15–24 years in HIV care. Public facilities with >90 AYH were shortlisted, and 10 facilities in each county were eventually selected based on accessibility (shorter distance from the town centers), previous working relationship, and not having a mental health intervention being tested in the HIV clinic. The 30 facilities selected included level 5 referral facilities, as well as level 4 and level 3 facilities – a categorization by the Ministry of Health based on resources available in the facility and catchment area.

A stratified randomization approach to balance for county and facility size (>150 AYH versus <150 AYH) was carried out. Matching was conducted for the 2 largest and better resourced facilities selected – one in Kisumu City and the other in Homa Bay – and for 2 facilities in Nairobi with >150 AYH. A total of 30 sites were randomized (10 per county): 15 to intervention and 15 to control arms.

### Provider selection, training and supervision

HIV clinic heads from the 30 facilities identified ~6 providers without specialist mental health training working closely with AYH, including clinicians, nurses, counselors, psychologists, and peer educators. The clinic heads at the intervention sites nominated 3 providers without mental health training from the 6 for PRO-ACT training. The training included: 1) Pre-training competency assessment using the World Health Organization’s ‘Enhancing Assessment of Common Therapeutic Factors’ (ENACT) rating scale [45], involving pre-developed and validated vignettes presented by trained standardized patient actors [39,46]; 2) 1-day ENACT competencies training; 3) 5-day PRO-ACT intervention training that included didactics, modeling, and small group practice of PRO-ACT skills with trainer feedback; 4) 1-day post-training ENACT competencies assessment; 5) 3 months of practice (with support supervision) of PRO-ACT in the facility with at least one AYH with mild-moderate psychological distress; and 6) 1 post-supervision ENACT competencies assessment. Notably, continuous PRO-ACT supervision will be provided during the trial period. The training and supervision was delivered by PRO-ACT-trained lay counselor experts as well as psychiatrists and psychologists from Kenya and Zambia. Supervision will be conducted virtually, with more intense supervision for initial cases (30 minutes before a session and 30 minutes-1 hour after) with intensity decreasing as providers increase in PRO-ACT adherence and competence. Providers in control sites will not be trained on PRO-ACT; however, the study team will conduct a sensitization meeting in each site before the trial on recruitment and enrollment, data collection, follow-up and referral procedures for AYH.

### Study procedures

#### A. Effectiveness.

**Fig 3:**
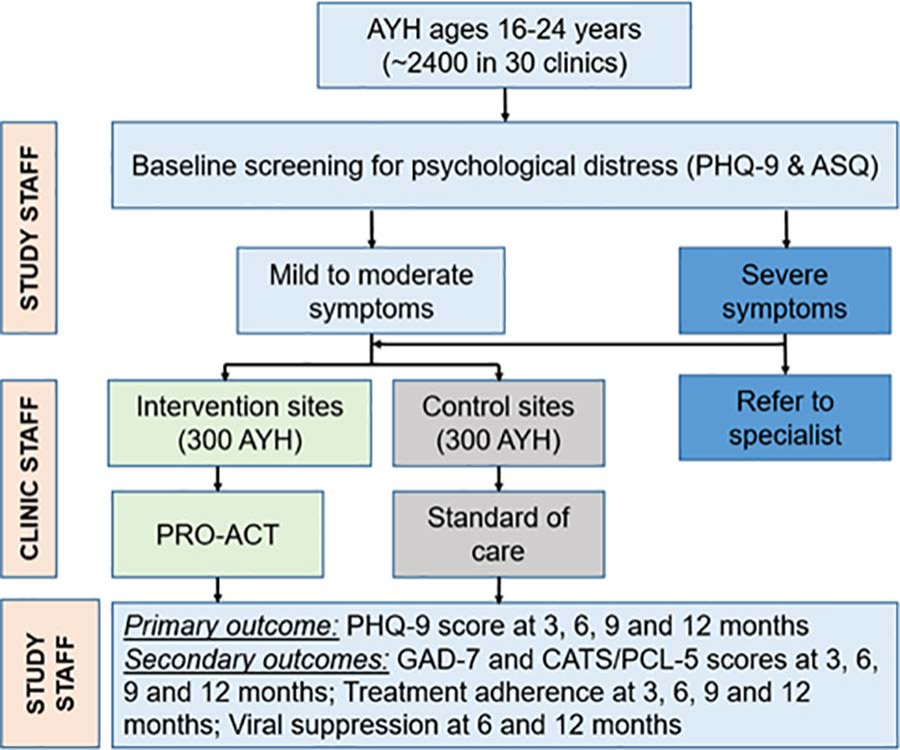
PRO-ACT study design.

The study aims to screen ~2400 AYH ages 16–24 years in the 30 facilities and enroll 300 AYH with psychological distress in each arm. In all participating sites, AYH will be screened for psychological distress by the study team. AYH who meet criteria for at least mild symptoms (Table 1) will receive PRO-ACT, while those in the control arm will receive standard of care interventions. Those with severe symptoms (Table 1) in both arms will be referred to mental health specialists. Follow-up assessments will be conducted at month 3, 6, 9 and 12 in both intervention and control arms (Fig 3).

**Table 1:** PRO-ACT effectiveness trial outcome measures.

Condition	Tool	Scoring criteria
***Primary outcome***		
Depression screening score	PHQ-9	No depression (0–4); Mild depression (5–9); Moderate depression (10–19); Severe depression (≥20)
***Secondary outcomes***		
Anxiety	GAD-7	No anxiety (0–4); Mild anxiety (5–9); Moderate anxiety (10–14); Severe anxiety (≥15)
Trauma symptoms	20-item CATS (<18 years) 20-item PCL-5 (18 + years)	≥21 is clinically relevant ≥31 is clinically relevant
Treatment adherence	Self-report on any antiretroviral therapy doses missed over the last 30 days	
Viral suppression (viral load <200 copies/ml)	Routine data abstraction	

*Recruitment and enrollment:* AYH in intervention and control clinics will be recruited during their routine clinic visits. A research assistant in collaboration with a designated clinic staff will approach all AYH ages 16−24 years during or immediately after triage. After a brief description of the study, AYH who wish to participate will be requested to provide oral consent to proceed with screening by the research assistant, which will include collection of data on sociodemographic characteristics, health care utilization, and the PHQ-9 [47]. Participants who report ever having suicidal thoughts or behaviors on PHQ-9 will be screened further using the ASK Suicide-Screening Questions (ASQ) tool [48] to determine urgency of need for referral to specialist mental health providers/institutions.

AYH with a PHQ-9 score ≥5 (psychological distress) or suicidality (ASQ score>1) will be scheduled for enrollment into the study and, if they are willing to proceed, will provide written informed consent. To participate in the trial, written informed parental consent, and assent by the adolescents ages 16−17 years will be requested. For adolescents ages 16 and 17 years who are unaccompanied to the clinic by a parent/ caregiver, a waiver of parental consent was granted by the ethics regulatory bodies. AYH will complete an enrollment questionnaire that will include socio-demographics, assessments of anxiety (7-item Generalized Anxiety Disorder scale), trauma (Child and Adolescent Trauma screen for AYH < 18 years or 20-item PTSD Checklist for DSM-5 for AYH ≥ 18 years) [49–51] and depressive symptoms (PHQ-9), alcohol and other substance use, HIV stigma, mental health stigma, healthcare utilization, social support, treatment adherence, and cost of seeking care.

*PRO-ACT delivery:* Enrolled AYH with mild or moderate or severe symptoms (following specialist evaluation) of psychological distress will be linked by the research assistant to the clinic staff trained to administer PRO-ACT at intervention sites. The provider and AYH will schedule at least 4 sessions at the convenience of the AYH, with an option of including a session with a caregiver or other support person if the AYH desires, within a period of 6 months. We anticipate that this will take into consideration the school term especially for young adults in boarding schools. While we anticipate PRO-ACT delivery largely for AYH with mild to moderate psychological distress, we will also offer PRO-ACT to AYH with severe symptoms after specialist evaluation, as access to any mental health intervention is often limited. Adverse events will be reported to the KNH-UoN ERC and NIMH in the annual reports. Severe adverse events will be reported to KNH-UoN ERC within 24 hours of the study being notified.

*Referral for specialist care:* Enrolled AYH in intervention and control sites with severe psychological distress, including suicidal thoughts and/or behaviors will be referred to a specialist mental health provider (psychiatrist, psychologist, nurse psychiatrist, or clinical officer psychiatry) available either in the facility or in another facility within the county. The research assistant will link the AYH with the HIV clinic’s clinical team who will make the appropriate referral and will provide support to the AYH to ensure the linkage to the specialist is completed. AYH in intervention clinics who are referred to specialists and receive a psychological intervention from the specialist will not receive PRO-ACT. They will however remain in the study and will complete surveys. Similar referral procedures will be instituted for those with severe symptoms during the follow-up visits.

*Follow-up:* AYH enrolled in the study will complete surveys administered by the research assistant at 3, 6, 9, and 12 months post-enrollment. The follow-up questionnaire will be similar to the enrollment questionnaire. The study will utilize the existing retention procedures for patients in each clinic including use of phone call and short messaging services reminders, and where available, home visits by retention officers or community health workers.

*Data collection and management:* Questionnaires will be completed using tablet-based REDCap tools [52], with the research assistant reading the questions aloud for the AYH and recording the responses. To collect sensitive data such as sexual behaviors, the AYH will have an option to complete the sections by themselves after a demonstration on how to navigate the tool. The study data team will also abstract routine clinical data from the electronic medical records (EMR) system in the facility or physical patient charts in cases where the clinical data has not been transcribed to the EMR. Viral load data will be abstracted from the national HIV viral load database. De-identified survey data will be downloaded weekly by a designated data manager, cleaned and stored in a password protected cloud storage – The University of Washington’s onedrive storage. The EMR data will also be stored in onedrive after abstraction and de-identification. Access to the data will be limited to only persons with approval from the Principal Investigator. The anonymized datasets will be uploaded to the NIMH data archive (NDA) every 3 months. All personal identifying information will be stored in a password protected link log, secured at REDCap. The link log will be destroyed after linkage of all datasets following completion of the study.

*Sample size calculations:* We anticipate about 25% of AYH (~20 participants) in each of the 30 clinics will have at least mild to moderate depression scores on the PHQ-9 and will be eligible to enroll in the study [53,54]. Based on this number of clinics, we calculated statistical power for three different treatment effect sizes (Cohen’s D [55], the standardized mean difference: 0.2, 0.3, and 0.4) under a cluster randomized design with an alpha value of 0.05. In addition to our expected enrollment per site, we also calculated power for 10 participants per site. S1 Table contains statistical power estimates for the effect sizes for three site level intraclass correlations (0.01, 0.03, and 0.05) [56], and the number of participants per site. For example, the second column of Table 3 contains the two statistical power estimates, 0.69 and 0.91, for a standardized mean difference of 0.3 with an intra-class correlation of 0.1 and sample sizes of 10 and 20, respectively. With a sample of 300 AYH per arm from 15 clinics each (~20 participants per site), the trial is adequately powered for any effects greater than 0.4, even with an ICC = 0.05.

**Table 3:** Determinants of implementation.

	How to measure	When to measure	Data source
**Self-efficacy**	Provider self-assessment of efficacy to implement PRO-ACT with providers	Baseline, early (month 3) and late (month 12) implementation	Survey with health care workers
**Organizational readiness**	Facility head and providers in the HIV clinic’s assessment of organizational readiness to implement PRO-ACT	Baseline, early (month 3) and late (month 12) implementation	Survey with health care workers
**Organizational citizenship behavior**	Facility head and providers in the HIV clinic’s own assessment of their, and the organizational citizenship readiness	Baseline, early (month 3) and late (month 12) implementation	Survey with health care workers
**Determinants of PRO-ACT adoption, acceptability and appropriateness**	Facility head and providers in the HIV clinic’s assessment of the factors contributing to the outcomes	Early (month 3) implementation	Qualitative interviews
**Determinants of reach, fidelity and maintenance**	Facility head and providers in the HIV clinic’s assessment of the factors contributing to the outcomes	Late (month 12) implementation	Qualitative interviews

#### B. Implementation.

*Implementation and client outcomes:* The assessment of implementation and service outcomes will be informed by the Reach, Effectiveness, Adoption, Implementation and Maintenance’ (RE-AIM) [57] and Proctor’s implementation outcomes frameworks [58]. We will quantitatively measure the following implementation outcomes: *Reach* of routine mental health screening by clinic providers [different from the screening study staff will carry out for eligibility] in intervention and control sites and *reach* of psychological interventions (PRO-ACT in intervention sites, other psychosocial interventions in control sites); *Adoption* of PRO-ACT in intervention sites; *Maintenance* of routine screening in intervention and control clinics and *maintenance* of PRO-ACT treatment practices in intervention sites; *Acceptability* and *appropriateness* of PRO-ACT in both intervention and control clinics at baseline (following the sensitization meetings with facility staff) and only in intervention clinics during follow-up. AYH’s *satisfaction* with services offered at the clinic will be measured at baseline and follow-up in intervention and control clinics. Providers will also rate their *satisfaction* with the training and supervision completed by the PRO-ACT trainers.

***Fidelity (adherence and competence):*** Psychotherapy supervision during the trial will include the assessment of fidelity of the implementation of PRO-ACT by providers in the intervention sites. Fidelity will be assessed at 2 levels: 1) provider self-report, using a checklist completed by the provider after each session on the PRO-ACT modules they covered, and 2) supervisor-report, wherein a supervisor (one of the PRO-ACT trainers) reviews the provider checklists and uses information from supervision sessions to rate provider *adherence* to PRO-ACT elements (score 1–6) and *competence* (score 1–6) for each session providers deliver to study participants (Table 2).

**Table 2:** Description of implementation outcomes as per Proctor’s IOF and Glasgow’s RE-AIM, measurement approach & frequency.

Implementation outcomes	Specific outcome & level of operationalization	How to measure	When to measure	Data source	Descriptive or comparative
**Reach**	Screening for depression. Level: patient	Intervention and control AYH receiving 1 + PHQ-9 within 12 months/ total AYH who have any visits	Baseline, month 3	Adolescent routine clinic records	Comparative
	Linkage to psychological intervention. Level: patient	Intervention and control AYH enrolled in psychological intervention/ total AYH with depression	Baseline, month 3, month 12	Adolescent surveys	
**Adoption**	Initiation of PRO-ACT intervention. Level: clinic	Intervention clinics who delivered PRO-ACT/ intervention clinics participating in PRO-ACT training	Month 3, month 12	Supervisor reports	Descriptive
	Initiation of PRO-ACT intervention. Level: clinic	Intervention providers who delivered PRO-ACT/ intervention providers participating in PRO-ACT training			
**Implementation/ fidelity**	Fidelity to PRO-ACT. Level: Provider and clinic	Proportion of providers implementing PRO-ACT with high adherence and competence. Assessed using a standardized checklist	Completed after each session with a patient	Supervisor-completed Likert score (also provider self-report)	Descriptive
**Maintenance**	Screening for depression. Level: patient	Intervention and control AYH receiving 1 + PHQ-9 within 12 months/ total AYH who have any visits	Month 12, endline	Adolescent routine clinic records	Comparative
	Linkage to psychological intervention. Level: patient	Intervention and control AYH enrolled in psychological intervention/ total AYH with depression		Adolescent routine clinic records and/ or adolescent surveys	
**Acceptability**	Acceptability of PRO-ACT	Acceptability of PRO-ACT, assessed using Acceptability of Intervention Measure (AIM) with providers	Baseline, early (month 3) month 6 and late (month 12) implementation	Survey with health care workers	Descriptive
**Appropriateness**	Appropriateness of PRO-ACT	Appropriateness of PRO-ACT assessed using Intervention Appropriate Measure (IAM) with providers	Baseline, early (month 3) month 6 and late (month 12) implementation	Survey with health care workers	Descriptive
**CLIENT outcomes**					
**Satisfaction with services**		Adolescent satisfaction with services received	Baseline, month 3, 6, 9, 12	Survey with enrolled adolescents	Descriptive
**Satisfaction with training**		Providers trained on delivering PRO-ACT	Post training	Survey with providers	Descriptive

*Determinants of implementation:* We will measure determinants of implementation quantitatively and qualitatively.

**Surveys with facility heads and providers:** We will measure *organizational readiness to change* [59], *organizational citizenship behavior* [60], *self-efficacy* [61] to implement psychological interventions in intervention and control clinics at baseline, and in intervention sites between month 3–6 of implementation (early) and at endline (Table 3).

**Qualitative interviews:** Between month 3 and 6 of the trial, we will conduct in-depth interviews (IDIs) with providers and facility heads to explore determinants of implementation for outcomes such as adoption, appropriateness, acceptability (early implementation outcomes) in intervention sites. Post-implementation, focus group discussions (FGDs) and IDIs with providers, AYH, and facility heads will focus on determinants of reach, fidelity, and maintenance (later outcomes) of screening, the PRO-ACT intervention, and other mental health interventions in control sites.

Focus group discussions will have 6–10 participants per site (separately for AYH and providers), while in-depth interviews will be conducted with between 1 and 3 providers and facility heads per site. The post-implementation IDIs will be complementary to the FGDs with AYH and providers to explore minority or poorly represented views in the FGDs. We will use constructs from available versions of the Consolidated Framework for Implementation Research (CFIR 1.0 and 2.0 and the LMIC adapted version of 1.0) [62,63] as a determinants framework to structure the interview guides to understand implementation determinants and contextual factors. We plan to assess select constructs within the domains of 1) Innovation, 2) Individuals (attention specifically to AYH and providers for AYH), 3) Inner setting, 4) Outer setting, 5) Implementation process, and 6) systems (focused on both HIV and mental health care). The constructs will be selected based on the investigative team’s experience during the implementation of this study and previous studies.

#### C. Costing and cost-effectiveness.

We will undertake a micro-costing of the resources needed for training, and the implementation activities in the intervention and control clinics. Four methods will be used to assess cost data: 1) micro-costing [64] to quantify resources used and unit costs, with data extracted from project expenditure and management records. 2) Time and motion logs conducted at baseline, month 6, 12 and 18 will apportion effort to the providers implementing the activities who divide time and effort among multiple roles. 3) The costs of specific program activities will be identified through structured interviews conducted at baseline, month 6, 12 and 18 with program officers; 4) a longitudinal patient survey conducted at baseline, month 3, 6, 9 and 12 with AYH to collect costs (including opportunity costs) of seeking care (Table 4).

**Table 4:** Costs and economic evaluation of the PRO-ACT intervention and its implementation.

Costs	When to measure	Data sources
***Program costs***		
Provider training, practice and supervision costs	Training, practice and supervision period	Study expenditure records
Provider time in training, practice and supervision	Training, practice and supervision period	Study training, practice and supervision records
Provider time offering intervention	Implementation period	PRO-ACT session tracking tool
Provider time offering services to AYH	Baseline, month 6, 12, 18	Time in motion studies
Research assistant time screening and linkage of AYH	Implementation period	Recruitment and enrollment logs
Recurring supplies and services, e.g., energy utilized in clinics, paper forms utilized for screening, and to track the intervention and referral	Training and implementation period	Survey with facility management, study expenditure records
Capital and equipment	Year 1 of implementation	Survey with facility management
Facility space	Year 1 of implementation	Measurement of HIV clinic spaces used for triage, clinical assessments, and counseling by a research assistant
***Patient costs***		
Direct patient costs: Cost of receiving services at the facility, transport and related costs to the facility	Year 1 of implementation Baseline, months 3, 6, 9 and 12	Survey with facility management AYH survey
Indirect patient costs: Missed opportunities at work or school to attend clinic, missed opportunities due to mental/other illness	Baseline, months 3, 6, 9 and 12	AYH survey

#### D. Implementation strategies.

This study will not formally test any implementation strategies [65]. However, we will utilize some strategies that have been shown to be effective in other studies to optimize implementation climate and fidelity.

We aim to optimize *implementation climate* by adopting a co-design framework [66] with key stakeholders in HIV and mental health care and research in Kenya to adapt the PRO-ACT intervention and its implementation to the adolescent and young adults HIV clinic context. A 2-day stakeholder workshop included representation of frontline providers and HIV clinic managers from the study sites, County and sub-County HIV and mental health leads/coordinators, national HIV and mental health programs leads. The workshop determined the additions and modifications required in the PRO-ACT modules to make it more responsive to AYH mental health needs, and preferences for providers to be trained and implement the intervention. Secondly, we conducted a brief training (1 day) for frontline providers and HIV clinic heads in intervention sites within the HIV clinic not directly implementing PRO-ACT, sub-County and County HIV and mental health program leads on the EQUIP competencies and the PRO-ACT intervention. Lastly, we will conduct dissemination meetings at each site and with the county-level stakeholders twice a year to present and discuss process and implementation outcomes.

We aim to optimize *implementation fidelity* through three approaches. First, PRO-ACT trainers deliver an active, skills-based training that includes not only didactics, but also trainer modeling, and provider practice with trainer feedback. Second, trainers will provide clinical supervision of providers trained to implement PRO-ACT (described under the intervention) throughout the study. Third, the providers, their HIV clinical care teams, and the study’s research assistants will convene continuous quality improvement (CQI) [67] forums. Through these discussions, the teams will identify implementation barriers and facilitators, identify and prioritize implementation strategies/innovations and test them over 2–4 weeks, and thereafter make decisions on whether to adapt, adopt or reject the strategy/innovation. Generally, the changes introduced and tested will not require additional resources from the study.

### Data analysis

#### Statistical analysis *for* effectiveness.

Primary analysis of PRO-ACT effectiveness will be conducted at 6- and 12-month follow-ups. The 6-month analysis provides an estimate of near-term treatment effect, while the 12-month analysis examines treatment effect sustainability. Both analyses will employ random coefficients models to account for clustered data structure. Our primary outcome is depressive symptoms, and anxiety and trauma symptoms are secondary outcomes. Models will include baseline scores as predictors, as well as county, demographics, school (boarding vs. day), and clinic type (young adults specific vs. integrated) as covariates. We will add provider competency to the final models to examine its role as a mediator of the treatment effect.

Secondary analyses will also include viral suppression as the outcome at 6- and 12-month; and will use a similar cluster data approach for the group randomized structure and strategy, but for a binary response (i.e., relative risk regression). The same covariates will be included in this analysis. Model assumptions will be checked for all analyses. No interim analysis are planned.

#### Analysis for implementation outcomes and determinants of implementation.

Descriptive analysis will be conducted for implementation and service outcomes. In addition, comparative quantitative analysis of reach and maintenance between intervention and control arms will follow a similar strategy to the mental health outcomes described above. Individual-level and provider-level analyses will require random coefficients models to accommodate clustering within site. In addition to testing whether reach of screening by routine program staff differs between arms, we will explore whether screening coverage by study staff has the unintended impact of decreasing screening by routine program staff.

We will assess whether: clinic infrastructure and staffing are associated with organizational and structural readiness, whether organizational and structural readiness are associated with early implementation outcomes (adoption, acceptability, and appropriateness), and whether early perceptions of acceptability and appropriateness (baseline) differ from later acceptability and appropriateness (3 month) using generalized linear models clustered on facility and on individual for the latter analysis.

FGDs and IDIs assessing determinants of early and late implementation will be audio-recorded, translated (as necessary), and transcribed. We plan to adopt a hybrid deductive-inductive approach to thematic analysis. Deductively, our analysis will be informed by the CFIR constructs and inductively based on the information provided by the study participants. Our analysis will follow the codebook approach to thematic analysis [68]. A team of two to three researchers will code selected transcripts to create a comprehensive codebook based in the CFIR codebook and conducting a thematic analysis. Two primary coders will independently code the remaining transcripts from IDIs and FGDs. Discursive meetings will be held with the coders and the research team to abstract the codes to formulate subthemes and themes.

#### Costs and cost-effectiveness analysis.

*Micro costing approach will be used to estimate the intervention cost, the cost will* be classified in one of four categories: a) personnel, b) recurring supplies and services, c) capital and equipment and d) facility space. Costs for capital items will be amortized on a straight-line basis over their expected useful life, and assuming no salvage value at the end of the useful life span on a capital asset. Facility space required for the intervention will be valued at the market rental rate. Output measures (denominator of the unit cost) include the numbers of patients receiving each type of study-supported services will be derived from patient files and study records.

Unit costs will be estimated based on relevant program and patient costs divided by outputs. We will assess the variation in unit costs across the study sites and identify the major determinants of that variation. We will compute the changes in unit cost over time as programs potentially achieve greater scale and administrative efficiency. We will also calculate the cost per additional AYH receiving mental health services. The unit costs of the full range of services provided will be calculated using a health system perspective as well as patients’ perspective.

We will estimate incremental cost-effectiveness, the added cost per case of depressive/anxiety/trauma symptoms resolved (health outcome). The numerator will reflect differences in the costs for the intervention arm and control arm. The denominator will represent the difference in the health outcome due to the clinical benefits of the intervention. The ratio is the incremental cost-effectiveness ratio (ICER) representing the change in cost per change health outcome across the intervention and control arm. Further, we will estimate the impact of uncertainty in inputs by conducting multi-way sensitivity analysis using Monte Carlo multi-variable simulations to estimate the confidence intervals associated with the base-case incremental cost-effectiveness ratios.

Dissemination of the results will be done to key stakeholders including participants and providers at the clinics, policymakers and other national stakeholders through local conferences and other meetings, and internationally through conferences, social media and publications.

## Discussion

Few studies have examined integration of mental health interventions beyond screening into HIV care for AYH, particularly in sub-Saharan Africa. Simms et al demonstrated that the addition of problem-solving therapy to the adherence support delivered by Community Adolescent Treatment Supporters (CATS) in Zimbabwe effectively reduced depressive symptoms among AYH [36]. Our study tests a brief, modular, transdiagnostic psychological intervention adapted to the AYH care setting in a resource-constrained context utilizing an innovative task-shifting approach. Methodological strengths include our attention to implementation processes including clear documentation of adaptations made to the intervention and a training and support supervision approach that is responsive to the HIV care context. The application of a hybrid type 1 effectiveness-implementation approach enables concurrent study of the impact of the intervention on mental health and HIV outcomes – which we are powered for, as well as implementation and cost outcomes, and determinants of implementation to inform future scale-up. Though not an objective of the study, we will collect data to explore the mediators and moderators of the effect of the intervention on mental health and HIV outcomes.

In determining the appropriate study design, the cluster randomized control trial (cRCT) is the most appropriate since the intervention would be delivered by existing health providers at the clinic level, in addition to their routine duties. An individual-level randomization faced a high risk of contamination since the same provider would offer the intervention in addition to the routine care. The sites selected provide reasonable diversity in clinic size, mental health resources availability and urbanicity, within regions with the highest burden of adolescent HIV in Kenya. Stratified randomization across region and clinic size in part ensures that clinic characteristics are balanced between intervention and control clinics.

The core intervention components are defined and will include a five-element intervention derived from the evidence-based for CBT interventions, and will be delivered in 4–6 sessions over a maximum 6-month period by adolescent HIV providers who are not mental health specialists. The training and support supervision of the providers as they deliver the intervention are additional components. The choice of providers to deliver the intervention (non-specialists) was informed by the increasing demand of task-shifted mental health interventions to integrate to primary health care and other routine services in hospital and community settings in resource-constrained countries. The focus of the various sessions was informed by stakeholders with extensive experience offering HIV and mental health services to AYH in Kenya. Though it is expected that each AYH receiving the intervention will present with unique challenges, the providers should be aware of common risk factors for poor mental health among AYH including HIV stigma, orphanhood, exposure to violence, poverty, and poor HIV treatment adherence.

The hybrid type 1 design will enable the measurement of implementation outcomes and determinants of implementation. However, though the study is utilizing evidence-based strategies such as continuous quality improvement, support supervision for providers, and dissemination strategies to optimize implementation climate, ownership and support, and implementation fidelity, we will not measure the individual or cumulative impacts of these strategies. In this study, we conducted intense training and consistent support supervision, which improves implementation fidelity and patient safety, but may not be feasible in a primary health care or program setting. Future studies evaluating task-shifted mental health interventions may evaluate different models of supervision, including their implementation and cost/cost-effectiveness to align to the realities of the HIV care set-up.

We anticipate that this intervention will improve functioning for AYH by reducing depressive, anxiety and trauma symptoms and enabling improved or sustained HIV treatment adherence and viral suppression, resulting in improved overall health. This has the potential for downstream effects on life achievement, including school and work performance, improved relationships, reduced risk of poor health behaviors, and reduced mortality through a reduction in suicidality and the sequelae associated with advanced HIV disease. The research is expected to have an impact on HIV service delivery by filling a critical gap in provision of holistic care that aligns with universal health coverage goals. Direct benefits will be to providers in intervention clinics who will be equipped with skills to offer a psychological intervention.

Our expectation is that the introduction of an intervention that helps AYH with common mental health conditions is likely to motivate providers to routinely screen AYH. Improved HIV outcomes for AYH may also reduce provider workload in longer-term since more AYH will be eligible for differentiated care with lengthier time between clinic visits. Further, this research will provide valuable lessons for the HIV community on the process and impact of managing co-morbidities to improve HIV treatment outcomes. Lastly, this study will conduct a cost-effectiveness analysis of the intervention and cost analysis of the implementation. These findings will help demonstrate whether/ how the costs align with plans for sustainability of HIV care in Kenya and similar resource-constrained settings.

## Supporting information

S1 Table Power estimates based on projected number of sites and individual adolescents and young adults from each site. (PDF)

S1 File Completed SPIRIT checklist. (PDF)

S2 File Version 1 of study protocol approved by the ethics and research committee. (DOCX)

S3 File Final version of study protocol approved by the ethics and research committee. (DOCX)
