# Supplementary material for: Integration of a brief, transdiagnostic psychological intervention in the care of adolescents and young adults with HIV in Kenya: Protocol for a cluster randomized clinical trial
Source: PLoS One. 2025 Jun 20;20(6):e0325374. doi: 10.1371/journal.pone.0325374 (PMC12180645; doi:10.1371/journal.pone.0325374)
Supplement: S1 Table — (PDF) [file pone.0325374.s001.pdf]

Supplementary table 1: Power estimates based on projected number of sites and individual adolescents and young adults from each site

	**Intra-class correlation**								
	0.01			0.03			0.05		
	**Effect size**			**Effect size**			**Effect size**		
**Number of participants per site**	**0.2**	**0.3**	**0.4**	**0.2**	**0.3**	**0.4**	**0.2**	**0.3**	**0.4**
10	0.37	0.69	*0.91*	0.33	0.62	*0.86*	0.29	0.56	*0.81*
20	0.6	*0.91*	*0.99*	0.48	*0.82*	*0.97*	0.40	0.73	*0.93*
