# Supplementary material for: Integration of a brief, transdiagnostic psychological intervention in the care of adolescents and young adults with HIV in Kenya: Protocol for a cluster randomized clinical trial
Source: PLoS One. 2025 Jun 20;20(6):e0325374. doi: 10.1371/journal.pone.0325374 (PMC12180645; doi:10.1371/journal.pone.0325374)
Supplement: S2 File — (DOCX) [file pone.0325374.s003.docx]

**Psychoeducation, Relaxation, PrOblem solving, Activation, Cognitive coping Therapy’ (PRO-ACT) for Adolescents and Youth in HIV Care**

Grant Number: 1R01MH133261

Principal Investigators:

Dalton Wamalwa, MBChB, MMED, MPH

Pamela Collins, MD, MPH

Muthoni Mathai, MBChB, MMED, PhD

Version Number: 1.0

July 11, 2023

**LIST OF ABBREVIATIONS**

| AIDS | Acquired ImmunoDeficiency Syndrome |
| --- | --- |
| ART | Antiretroviral Therapy |
| AYHIV | Adolescents and Youth with HIV |
| CATS | Child & Adolescent Trauma Screen |
| CMD | Common Mental Disorders |
| COVID-19 | CoronaVirus Disease of 2019 |
| cRCT | Cluster Randomized Control Trial |
| DALY | Disability Adjusted Life Year. |
| EQUIP | Ensuring Quality in Psychological Support |
| GAD-7 | Generalized Anxiety Disorder-7 |
| HIV | Human Immunodeficiency Virus |
| LTFU | Loss To Follow Up |
| NASCOP | National AIDS andSTI Control program |
| PCL-5 | Post Traumatic Stress Disorder Disorder Checklist 5 |
| PHQ-9 | Patient Health Questionnaire-9 |
| PRO-ACT | Psychoeducation, Relaxation, problem solving, Activation, Cognitive Therapy |
| PTSD | Post Traumatic Stress Disorder |
| TF-CBT | Trauma Focused Cognitive Behavioral Therapy |
| UHC | Universal Health Coverage. |
| WHO | World Health organization |

**PROTOCOL SUMMARY**

| **Title:** | Psychoeducation, Relaxation, PrOblem solving, Activation, Cognitive coping Therapy’ (PRO-ACT) for Adolescents and youth in HIV Care |
| --- | --- |
| **Objective:** | This study aims to adapt and evaluate a brief transdiagnostic psychological intervention (PRO-ACT) for adolescents and youth with HIV (AYHIV) for delivery by non-specialists; and determine contextual implementation factors and cost of implementation to inform scale-up. |
| **Aims:** | Aim 1: Adapt and optimize the delivery of PRO-ACT for HIV clinics serving AYHIV ages 16-24 years in Kenya  Aim 2: Evaluate the effectiveness and implementation of PRO-ACT for AYHIV ages 16-24 years in a cluster randomized trial  Aim 3: Determine cost of implementation for PRO-ACT training, delivery, and implementation process |
| **Methods** | Aim 1: Adaptation of PRO-ACT training and delivery will be conducted through a stakeholder-led process.  Aim 2: Effectiveness-implementation hybrid 1 cluster randomized controlled trial (cRCT). Optimization of PRO-ACT delivery will be done in 15 intervention sites after training of the non-specialist providers. We will use continuous quality improvement over a 6-months period.  Aim 3: Economic evaluation taking into account costs at various levels |
| **Population:** | Stakeholders: Policy makers, providers, adolescents and youth with HIV, caregivers, researchers  Health care workers: Currently employed in HIV care services  Patients: Adolescents and youth with HIV ages 16-24 years |
| **Number of Sites:** | 30 sites in Nairobi, Kisumu and Homa Bay counties in Kenya |
| **Study Duration:** | 5 years |
| **Outcomes** | Aim 1: Provider training   - Non-specialist providers’ competence to deliver PRO-ACT - Non-specialist providers’ satisfaction with PRO-ACT training   Aim 2: cRCT   - Primary Effectiveness Outcome: Reduction in depressive, anxiety and trauma symptoms among AYHIV - Secondary Effectiveness Outcome: Viral suppression among AYHIV - Implementation Outcomes: Reach, adoption, fidelity / implementation, maintenance, acceptability of PRO-ACT - Service Outcomes: Satisfaction with services by AYHIV   Aim 3: Economic evaluation   - Cost of PRO-ACT provider training and implementation - Incremental cost-effectiveness: net added cost per case of depressive/anxiety/trauma symptoms resolved (health outcome) |
| **Primary analysis** | Effectiveness of PRO-ACT will assessed using 6-month analysis to provide an estimate of the near-term treatment effect, and at 12 months to examine treatment effect sustainability. The analyses will employ random coefficients models to account for clustered data structure, and compare outcome means separately (depression, anxiety, trauma) between the treatment and control groups. |

1. **BACKGROUND INFORMATION AND SCIENTIFIC RATIONALE**

**1a. Background Information**

Mental health is defined as a state of mental well-being that enables people to learn, work, cope with life stressors and contribute to their community.^1^ If an individual is unable to achieve this equilibrium, they face a challenge in using their abilities in accordance with accepted societal values.^2^ Such individuals find difficulty in recognizing, expressing and modulating their own emotions and use of basic cognitive and social skills in problem solving and coping with adverse life events. Over 900 million people around the world were living with a mental disorder in 2019, with anxiety and depressive disorders the most common.^3^ Due to the COVID-19 pandemic, this number rose significantly in one year, 2020 recording a 28% increase.^4^

Close to 25% of youth worldwide suffer from Common Mental Disorders (CMD) which include depressive and anxiety disorders, These disorders differ from fear and stress that any person can experience at some moment in life.^5^ The vast majority of these youth reside in resource limited settings.^6^ Mental health screening may help in early detection and intervention which allow for more effective pathways to be taken long before the problems commence or worsen.^7^ It also allows for tailoring interventions to specific populations.^8-11^. Transition from childhood to adulthood has registered a peak in incidences of mental health disorders, in five people; at least one has experienced clinically relevant mental health problems before attaining 25 years.^12^

Guidelines on promoting mental health among adolescents by the World Health Organization identify six main areas of focus among them, psychosocial interventions for Adolescents and Youth with HIV/AIDS (AYHIV) to prevent mental disorders, self-harm and other risky behaviours.^13^ Kenya has developed policies and guidelines for mental health care. The constitution of Kenya 2010 under chapter 48 outlines the rights of patients with mental health problems and gives modalities of establishing institutions that manage mental health in the national and devolved governments.^14^ National guidelines for provision of adolescent youth-friendly services in Kenya 2005 focused on the general wellbeing and reproductive health of youth with a very small portion on mental health.^15^ These guidelines were revised in 2016 with a goal of improving availability, accessibility and quality of healthcare for the adolescent and youth^16^ still with a major bias towards reproductive health and a small component of mental health.

During the COVID-19 pandemic, a comprehensive guideline on mental health and psychosocial support was developed by the ministry.^17^ The Ministry of Health Mental Health Policy 2015-2030 was developed with the following objectives; align mental health services to the constitution of Kenya, national and global health agenda; address the mental health challenges and trends and mitigate their burden; integrate mental health services within the Kenya Essential Package for Health (KEPH); and promote and respect rights of persons with mental health challenges.^18^

Kenya has 1.4 million people with HIV/AIDS, with a national prevalence at 4.9%, prevalence in women being 6.6% while men being 3.1%. The gender disparity among the youth is higher.^19, 20^ The top five high-prevalence counties are Homa Bay (~20%%), Kisumu (~18%), Siaya (~15%), Migori (~13%) and Busia (~10%)^20^. These counties and Nairobi County also have the highest burden of HIV among adolescents and youth^20^. New infections in Kenya are on an increase, for instance, young people (15–24 years old) contributed an estimated 12,000 infections (>50% of all adult infections) in 2021-2022.^20^

**1b. Significance and rationale**

*Mental disorders are a major cause of morbidity among adolescents and youth with HIV (AYHIV).^21^* Globally, approximately 3.5 million young people (age 15-24 years) live with HIV; 60% are girls, the majority (>80%) in sub-Saharan Africa (SSA).^22,23^ Improved survival of children due to successful roll-out of antiretroviral therapy and new infections in adolescence contribute to rising numbers of AYHIV. Kenya is a high HIV-burden country with nearly 150,000 young people living with HIV and 14,000 new infections annually.^24^ Vulnerability to HIV and mental health conditions intersect in adolescence and youth, a sensitive period of neuropsychological and social development during which individuals seek greater autonomy and initiate sexual activity.^25,26^ HIV is associated with mental health problems and decreased quality of life for adolescents and young people.^27-30^ AYHIV suffer from depression and anxiety disorders as they adjust to the diagnosis, adapt to life with a chronic, life-threatening disorder, experience stigma, and manage disease progression.^25, 31-33^ Several systematic reviews highlight the substantial burden of mental health problems facing AYHIV.^32,34-38^ Recent reviews report prevalence of any psychiatric disorder near 25%, anxiety 25-44%, and depression 25-50% among AYHIV. Studies in Kenya have documented similarly high rates of psychiatric morbidity among AYHIV, (prevalence of 50% for any psychiatric illness and depression).^39,40^ AYHIV rates exceed prevalence in the general population.^41-45^

*Modifiable correlates of mental disorders among AYHIV are well known and can form targets for intervention.*^32^ Community level factors (HIV-stigma and violence), family level factors (poverty, quality of parenting, and caregiver mental health) and HIV-related factors (rapport with healthcare providers and duration on ART) directly influence mental health.^46-50^ Older and behaviorally-infected HIV adolescents and youth have the highest rates of mental disorders and the lowest level of support from the health system due to health provider bias.^51^ Our group showed that 22% of Kenyan adolescents receiving care in a comprehensive HIV clinic in Nairobi changed homes in the past year and 30% missed a meal involuntarily, reflecting unstable social support and widespread poverty.^39^ As we pursue interventions to address these risk factors,^52-54^ there is an immediate need to address their effects on mental health.

*Mental disorders increase non-adherence to HIV care and viral non-suppression, which affect quality of life and mortality risk.*^55-58^ AYHIV with psychological distress, depression, anxiety and post-traumatic stress consistently have lower ART adherence, poor retention in care, and viral load non-suppression compared to those without these disorders.^59-61^ Depressive symptoms disrupt daily activities, reduce concentration, foster non-adherence, and increase poor HIV treatment outcomes. Together, HIV disease and depression weaken school performance,^62^ which can reduce future economic prospects. Ideally, adolescent mental health care helps prevent harmful social consequences in adulthood.^63,64^

*Effective depression treatments exist and have been linked to improvement in ART adherence.*^65-67^ Use of psychotherapy in addition to medication effectively improves depression and treatment adherence among AYHIV.^68^ In a recent meta-analysis, the odds of adhering to HIV care was 83% higher for those receiving care/interventions for depression or psychological distress.^65^ Integrating mental health and HIV services can help prevent new HIV infections and improve the health of people living with and affected by HIV by increasing access to coordinated care for people with complex conditions, enhancing provider communication, reducing stigma associated with seeking specialty care, and removing practical challenges of multiple care visits.^69-71^ Collaborative care models and task-sharing with peers, lay health workers, adherence counsellors, or other community-based human resources are useful strategies for administering evidenced-based psychosocial treatments in HIV affected communities in settings where mental health specialists are scarce.^72-77^

*SSA needs more practical, validated, scalable strategies to treat mental disorders among AYHIV.*^78,79^ Global targets require 90% of people with HIV linked to people-centered and context-specific integrated services by 2025.^80^ International and Kenyan HIV care guidelines call for psychosocial support for AYHIV, screening for depressive symptoms, psychotherapy for mild-moderate symptoms, and referral to mental health specialists for severe symptoms.^81-85^ However, weak systems, a paucity of competent providers,^78^ an overstretched workforce,^86-88^ and lack of a clear interface between HIV clinics and mental health specialists prevents guideline implementation.^89^

*Health care in Kenya is devolved with different mandates for the national versus county health systems.* The Kenya national Ministry of Health (MOH) is primarily tasked with policy development (including adolescent and mental health policies) and administrative oversight over vertical programs like the HIV program, the National AIDS/STI Control program (NASCOP). The national MOH also manages large tertiary health institutions including the major mental health hospitals located in large cities. Most Kenyans, however, seek care in county-level hospitals, managed by 47 different county administrations, charged with clinical service delivery, in-service provider training and implementation of programs^90^. A presidential taskforce on mental health in Kenya noted the glaring deficiencies in mental health care competency among providers in the county health facilities, and the need to devolve mental healthcare.^91^ This decentralization is a key component of universal health coverage (UHC), which Kenya is in the process of adopting.^92^

*Apart from a few medium to large well-funded HIV clinics in urban settings, integration of mental health within HIV services in Kenya is largely non-existent.* Kenya has insufficient mental health workers in public service for its population (100 psychiatrists, 500 psychiatrist-nurses), and the majority deliver specialist care in large facilities. Sub-county HIV clinics, where many AYHIV are managed, completely lack trained mental health personnel. In a typical HIV clinic AYHIV are not routinely evaluated for mental illness, but screening with the PHQ-9 may occur among AYHIV with non-suppressed viral loads. This may not lead to further assessment or referral due to inadequate clinician knowledge of the tool or lack of interface with specialists.

*The HIV program in Kenya offers an excellent platform to develop and study integration models for mental healthcare in routine care.* The HIV program in Kenya envisions integrating non-communicable disease care into routine HIV care, suggesting some readiness to accept innovations in this area. Kenya was among the first SSA countries to scale up comprehensive prevention of mother-to-child transmission,^93,94^ pediatric index-case testing,^95^ HIV self-testing and pre-exposure prophylaxis,^96^ and use of dolutegravir-based regimen.^97^ Additionally, the HIV program has experience with in-service provider training for patient-centric services^98,99^ and interventions involving task-shifting,^100^ making service provision more accessible to patients. Most HIV services are offered by non-specialist providers, including general medical officers, clinical officers, nurses and counselors without specialized training in psychology.^86^ Peer educators play a major role in care, especially treatment support for AYHIV.^101^ However, there are gaps that are amenable to targeted interventions. Routine screening and management of mental disorders is limited to a few well-resourced tertiary centers with trained mental health personnel.^91,102,103^ The scheduling of clinic visits for AYHIV to accommodate the school calendar may affect the implementation of mental health interventions.^104,105^ Our prior works shows that school-going youth attending boarding school make up 20% of AYHIV and they can only access mental health care on term and mid-term breaks. To address these challenges, there is an urgent need to integrate mental health services into the routine clinic visits for *all* AYHIV so that assessment and management of mild to moderate disorders and referral for severe symptoms occurs. To this end advances in modular, transdiagnostic psychological interventions hold great promise.

*Transdiagnostic interventions may be preferable to diagnosis-specific interventions*^106^ *in HIV care.* People with depressive, anxiety, or trauma disorders rarely present with ‘pure’ symptom criteria that align with a single diagnosis; rather, the conditions frequently co-occur.^107^ Thus, single-disorder-focused treatments may not match the more common clinical need.^108^ A meta-analysis of studies targeting youth for either depression or anxiety found a “cross over effect”: interventions targeting depression also reduced anxiety and vice versa, strengthening the case for transdiagnostic interventions.^109^

The Common Elements Treatment Approach (CETA) is a transdiagnostic psychotherapy developed by Dorsey and colleagues (Co-I on this proposal) that is based on common evidence-based treatments for depression, anxiety, trauma and stress-related disorders.^110,111^ CETA includes important advancements, but requires 8-12 sessions, making it difficult to integrate into HIV care for adults and school-going adolescents. A recent study in Mozambique using CETA for adults with HIV found that 80% achieved >50% reduction in symptoms in 6 or fewer sessions.^70,112^ The setback however was that only 14% completed treatment. This suggests a need for briefer transdiagnostic interventions like **‘Psychoeducation, Relaxation, PrOblem solving, Activation, Cognitive coping Therapy’ (PRO-ACT)** a brief trans-diagnostic intervention developed and piloted in Kenya among youth that can avoid high attrition due to length.^113^ Studies among adolescents and young adults in high-income countries have found brief psychological interventions effective.^114^ Only one study in an African country suggests that brief interventions for AYHIV may be preferable.^115^ Our pilot of PRO-ACT supports this finding. In the absence of adequate evidence on what works in integrated HIV care, the proposed study meets the need for the evaluation of brief transdiagnostic interventions that are effective and applicable for HIV services in low resource settings.^78^ This intervention will be particularly advantageous for adolescents and youth in school who cannot sustain lengthy programs.

*Quality of mental health care can be enhanced by assessing provider competency to target training and supervision.*^116^ The Enhancing Assessment of Common Therapeutic factors (ENACT) rating scale,^117,118^ used in the World Health Organization’s Ensuring Quality in Psychological Support (EQUIP) provider training platform,^119^ enables competency assessment by specialist or non-specialist (and peer) supervisors. ENACT consists of 15 core competencies, i.e., common factors among psychotherapies that influence client outcomes positively, and has been applied primarily in LMICs, where innovations in deployment of non-specialist mental health providers are more prevalent, given health system and community needs.^120^

1. **OBJECTIVES**

**2a. Study Objective**

This study aims to adapt and evaluate a brief transdiagnostic psychological intervention (PRO-ACT) for adolescents and youth with HIV (AYHIV) with delivery by non-specialists; and determine contextual implementation factors and cost of implementation to inform scale-up.

**2b. Specific Aims**

**Aim 1: Adapt and optimize PRO-ACT for HIV clinics serving AYHIV ages 16-24 years in Kenya**

Approach: With input from stakeholders, we will adapt and pilot PRO-ACT training and delivery for AYHIV in HIV clinics. Stakeholders include HIV/mental healthcare workers, policymakers, facility staff, and AYHIV. In collaboration with stakeholders, we will tailor implementation for the context (e.g., the specific cadre and when in the clinic visit mental health screening and management will be done).

**Aim 2: Evaluate the effectiveness and implementation of PRO-ACT for AYHIV ages 16-24 years.**

Approach: We will conduct a hybrid 1 effectiveness-implementation cluster randomized control trial in 30 HIV clinics. We will select and randomize 30 adolescent HIV clinics in Nairobi, Kisumu, and Homa Bay counties, and train non-specialist providers in 15 intervention sites. We will optimize the delivery of PRO-ACT in the intervention sites in a 6-month pretrial period using continuous quality improvement processes. Primary effectiveness outcome: Reduction in depressive, anxiety and trauma symptoms among AYHIV. Secondary outcome: Viral suppression among AYHIV. Implementation outcomes: Compare reach and describe adoption, fidelity / implementation, maintenance, acceptability. Identify determinants of reach, fidelity, and maintenance. Service outcomes: satisfaction of training by providers and satisfaction with services by AYHIV.

*Hypothesis: The intervention will result in a significantly larger reduction of depressive, anxiety and trauma-related symptomatology over 12 months compared to the standard of care. Implementation of the intervention will improve the reach and fidelity in mental health screening and management for AYHIV.*

**Aim 3: Determine cost and cost-effectiveness of implementation for PRO-ACT training, delivery, and implementation process.**

Approach: We will conduct a time-driven activity-based costing of the intervention’s implementation strategies (training and implementation in clinics) within the 30 clinics in Kenya participating in the study to estimate the implementation costs from a patient and health system perspective. We will in addition utilize the health outcome data based on the health outcome of the intervention’s implementation in the 30 sites to estimate cost effectiveness or the intervention.

*Hypothesis: There will be an increase in costs related to the training and time for providers offering mental health services and a reduction in projected costs related to poor HIV-related and mental health. The incremental cost effectiveness ratio of the interventions will be positive.*

1. **STUDY OUTCOMES**

**3a. Primary outcomes**

Aim 2: Cluster randomized controlled trial (cRCT)

The primary outcome for this study is **Mean change in depression scores, anxiety scores and trauma scores** measured longitudinally (5 measures) over 12 months as follows:

- Depressive symptoms: Using the Patient Health Questionnaire (PHQ-9)
- Anxiety symptoms: Using the Generalized Anxiety Disorder-7 (GAD-7)
- Trauma symptoms: Using the Child and Adolescent Trauma Screen (CATS) for AYHIV <18 years and the PTSD checklist for DSM-5 (PCL-5) for AYHIV ≥18 years

**3b. Secondary outcome**

Aim 2: Viral suppression among AYHIV (secondary effectiveness outcome)

- Measured from abstracted clinic records and defined as viral load of <200 copies/ml.

Primary and secondary effectiveness outcomes are summarized in table 1

| **Table 1: Primary and secondary effectiveness outcomes** | | | | | |
| --- | --- | --- | --- | --- | --- |
| **Condition** | **Tool** | **Number of items** | **Validated age group** | **Time points for assessment** | **Scoring criteria** |
| ***Primary outcomes*** | | | | | |
| Depression | PHQ-9/M | 9 items | 12+ years | Baseline, 3, 6, 9, 12 months | No depression (0-4)  Mild depression (5-9)  Moderate depression (10-19)  Severe depression (≥20) |
| Anxiety | GAD-7 | 7 items | 12+ years | Baseline, 3, 6, 9, 12 months | No anxiety (0-4)  Mild anxiety (5-9)  Moderate anxiety (10-14)  Severe anxiety (≥15) |
| Trauma symptoms | CATS  PCL-5 | 20 items  20 items | 7-17 years  18+ years | Baseline, 3, 6, 9,12 months | ≥21 is clinically relevant  ≥31 is clinically relevant |
| ***Secondary outcomes*** | | | | | |
| Viral suppression | N/A | N/A | N/A | Baseline, month 6, month 12 | <50 copies/ml: Undetectable  <1000 copies/ml: Suppressed |

- Aim 2: Implementation and service outcomes are summarized in table 2

| **Table 2. Description of implementation outcomes as per Proctor's IOF and Glasgow's RE-AIM, measurement approach & frequency** | | | | |
| --- | --- | --- | --- | --- |
| **Implementation outcomes** | **Specific outcome** | **How to measure** | **When to measure** | **Data source** |
| **Reach** | Screening for anxiety and depression | Proportion of AYHIV in intervention vs control clinics screened using the PHQ-2/PHQ-9 tool out of total AYHIV who should be screened | Early (month 3) and late (month 12) implementation | Patient records |
|  | Linkage to psychological intervention | Proportion of AYHIV in intervention vs control enrolled in the psychological intervention among those diagnosed with anxiety and/or depression |  |  |
| **Adoption** | Initiation of PRO-ACT intervention | Proportion of intervention clinics that participated in the PRO-ACT initial training and elected to deliver PRO-ACT | End-line | Study records |
| **Implementation / fidelity** | Fidelity to PRO-ACT | Proportion of intervention clinics implementing PRO-ACT with high fidelity. Assessed using a standardized checklist and self-report | Early (month 3) and late (month 12) implementation | Checklist |
| **Maintenance** | Screening for anxiety and depression | Proportion with sustained screening practices (% of AYHIV with a routine PHQ-2 at each visit) | Late (at month 12) implementation covering up to 12 months of adolescent treatment | Routine clinic patient records |
|  | Delivery of PRO-ACT intervention | Proportion with sustained treatment practices (% of AYHIV eligible for PRO-ACT with any PRO-ACT modules completed at each visit) |  | Checklist & patient records |
| **Acceptability** | Acceptability of PRO-ACT | Acceptability of PRO-ACT among intervention clinics, assessed using Acceptability of Intervention Measure (AIM) | Early (month 3) and late (month 12) implementation | Survey with health care workers |
| **SERVICE outcomes** | | | | |
| **Satisfaction with services** |  | Adolescent satisfaction with services received | Late implementation (at month 12) | Survey with enrolled adolescents |
| **Competency assessment** |  | ENACT assessment for providers | Pre-training, post-training, post-supervision | ENACT through EQUIP platform |
| **Provider satisfaction** |  | Provider satisfaction with the training | Post-training | Survey with providers |

Aim 3: Economic evaluation

This aim will assess the following measures:

- Cost of PRO-ACT provider training and implementation
- Incremental cost-effectiveness: net added cost per case of depressive/anxiety/trauma symptoms resolved (health outcome)

*Additional exploratory outcomes*

- Cost per additional adolescent or youth with HIV with ≥80% adherence
- Cost per additional adolescent or youth with HIV virally suppressed

**4.** **STUDY DESIGN**

This study uses a hybrid type 1 cluster randomized clinical trial to evaluate the effectiveness of the PRO-ACT intervention to reduce depressive, anxiety and trauma-related symptoms, and study the implementation and cost of the intervention. A cluster RCT is suitable as the intervention will be implemented at the clinic and provider level rather than exclusively at the individual AYHIV level. The adaptation and optimization phase will ensure that the intervention is relevant and suitable for delivery in the HIV clinic, with all relevant providers in the intervention clinics trained on PRO-ACT.

**5. STUDY ENROLLMENT AND WITHDRAWAL**

**5a. Study sites**

The stakeholders workshop and the subsequent refinement of PRO-ACT will be coordinated at a centralized site in Nairobi. The pretrial contextual adaptation of PRO-ACT, and the cluster randomized controlled trial (cRCT) will be conducted in 30 HIV clinics selected from two counties – Nairobi, Kisumu and Homa Bay counties in Kenya.

**5b. Study population**

The stakeholders workshop will involve policymakers, frontline providers, researchers, adolescents and youth with HIV and their caregivers. Non-specialist providers in adolescent HIV clinics selected to participate in the study and randomized to intervention sites will be trained on PRO-ACT and will administer the intervention. Providers in both intervention and control sites will also participate in the implementation of the study. Adolescents and youth ages 16-24 years in the selected intervention and control clinics will be eligible to participate in the study.

**Justification for involvement of adolescents:** In sub-Saharan Africa, adolescents living with HIV are at high risk for poor mental health and poor treatment outcomes. This study aims to address adolescent mental health and HIV care. The data from this study will be used to assess the effectiveness of the psychological intervention, its implementation and cost effectiveness in adolescent HIV care settings.

**5c. Participant inclusion criteria**

Eligibility is summarized in table 3. Participants in the stakeholders workshop will be adults except for the 2 minors (<18 year olds) in the group representing AYHIV. Non-specialist providers ages 18 years and above employed in the selected adolescent HIV clinics will be eligible for training (if in intervention sites) and other study procedures in both intervention and control sites. All AYHIV ages 16-24 years in the selected sites will be eligible to participate in the study (including abstraction of their clinic data and completing surveys), while those with mild-moderate symptoms of psychological distress following screening in the intervention sites will be eligible for the PRO-ACT intervention. The HIV clinics will be selected from among the public facilities in Nairobi, Kisumu and Homa Bay counties with at least 100 adolescents and youth ages 10-24 years in care, and no other psychological intervention being tested in the clinic.

**5d. Site/Participant exclusion criteria**

A facility may be excluded if there are barriers to conducting provider training or data collection/ abstraction. Individuals will be excluded if any study procedure would put them at an increased risk or if their compliance with study procedures is not possible. Adolescents below 16 years will be excluded since we expect that majority of them will require additional interventions targeting caregivers since they have not achieved independence in care, and have not been fully disclosed to their HIV status. This younger age group therefore requires a separate study with an intervention adapted to their needs.

| **Table 3: Populations involved in the study** | | | |
| --- | --- | --- | --- |
| **Aim** | **Design** | **Approach/strategy and data source** | **Participant numbers and inclusion characteristics** |
| AIM 1: Adapt and optimize the training on PRO-ACT and the intervention delivery | Cross-sectional | Stakeholder workshop | 40 stakeholders including policymakers, frontline providers, researchers, representatives of adolescents and youth with HIV (AYHIV) and other community members |
|  |  | Continuous quality improvement (CQI) of PRO-ACT delivery in 15 public HIV clinics selected for the cluster randomized trial as intervention sites | Approximately 120 providers ≥18 years old at 15 intervention clinics (up to 6 per site trained on the PRO-ACT intervention); and up to 6 AYHIV age 16-24 years per site. |
| AIM 2: Test effectiveness of the PRO-ACT intervention | Cluster randomized trial | 30 public HIV clinics each with at least 100 AYHIV | AYHIV age 16-24 years attending public HIV clinics. We expect ~2400 AYHIV will be screened and ~300 AYHIV with mild to moderate psychological distress will be followed-up in each arm, with the ~300 in the intervention clinics receiving the intervention |
|  |  | Survey assessing provider satisfaction with training | Up to 90 trained providers ≥18 years old at 15 intervention clinics (up to 6 per site) |
|  |  | Surveys | All AYHIV (~2400) ages 16-24 years in the 30 participating clinics will be screened using mental health assessment tools in a survey.  The ~300 AYHIV followed up in each arm will complete follow up surveys every 3 months |
|  |  | Routine clinic data | Clinic notes and viral load results will be abstracted from the charts and electronic medical records repositories for all AYHIV (~2400) ages 16-24 years in the 30 clinics |
| AIM 2: Assess implementation and service outcomes |  | FGDs with providers | 30 with 7-10 participants each from the intervention and control facilities ≥18 years old |
|  |  | IDIs with providers | 6 IDIs with providers from each participating clinic |
|  |  | Competence assessments using the ENACT tool | Up to 90 trained providers ≥18 years old at the 15 intervention clinics |
|  |  | Quantitative surveys with AYHIV | ~600 AYHIV involved in the intervention |
|  |  | Routine clinic records for AYHIV | All AYHIV in the 30 clinics ages 16-24 years |
|  |  | Checklist in the patient chart | All AYHIV (~2400) AYHIV 16-24 years in the study sites |
| Aim 3: Conduct an economic evaluation of the intervention |  | Time in motion studies for provider time | Providers in the 30 adolescent HIV clinics |
|  |  | Interviews with program officers with information on program administration | 30 program officers in the HIV clinics |
|  |  | Quantitative surveys with AYHIV | ~600 AYHIV involved in the intervention |

**5e. Strategies for recruitment and retention**

**Aim 1:** *Adaptation of PRO-ACT by stakeholders:* We will leverage the expertise and professional networks the study team has within the mental health and adolescent HIV care domains to purposively select stakeholders who will be involved in the workshop adapting the PRO-ACT training for healthcare providers. We will purposively select up to 40 stakeholders who will include:

1. Representatives from the Ministry of Health Departments of Mental Health and Adolescent Health

2. Representatives from the County health departments, specifically those overseeing the adolescent HIV program and mental health programs

3. Representatives from the National AIDS and STI Control Program (NASCOP) overseeing the adolescent HIV program

4. Representatives of HIV care implementation partners (non-governmental organizations supporting the adolescent HIV program)

5. HIV and mental health researchers and academics from various universities, NGOs and teaching hospitals in Kenya and the University of Washington

6. Representatives from regional HIV Technical working Groups

7. Frontline healthcare providers working in HIV clinics

8. Youth living with HIV nominated by the youth-led advocacy groups

9. Caregivers of adolescents and youth living with HIV nominated by advocacy groups

10. Adolescent and youth mental health advocates in Kenya

The stakeholders will be reached in-person or via phone call or email to be informed about the study. They will then receive a formal invitation to the workshop. We will provide them periodic updates of the study to maintain their engagement in the project. They will receive reimbursement for the time used in study activities.

**Aim 1:** *Provider training:* Providers in intervention clinics who will receive the PRO-ACT training will be purposively selected by the HIV clinic head (~6 per clinic). They will be providers of different cadres (clinicians, nurses, counselors and peer educators) working directly with youth. Their contact information will be collected by the study staff from the clinic head, and they will provide consent to participate in the training and subsequent study procedures.

**Aim 2:** *Supervision and optimization of PRO-ACT delivery in intervention sites:* Each trained provider (up to 6 per intervention site) will screen youth receiving care at their clinic ages 16-24 years for depression, anxiety or trauma symptoms during their routine clinic or antiretroviral treatment refill visits. Each provider will be allocated at least one AYHIV with mild to moderate symptoms of psychological distress. The AYHIV will be consented and enrolled into the study to receive the PRO-ACT intervention, where over a 3-6 month period, they will attend sessions depending on their availability. They will be reminded of their scheduled sessions via a phone call or text message, a week and a day before the session. The sessions will be supervised by a PRO-ACT trainer (psychologist), with a debriefing between the supervisor and provider also done after the session. The providers will hold bi-weekly meetings to optimize the intervention. The providers will receive reimbursement for the time they perform study activities, while the AYHIV will be reimbursed for the cost to get to the clinics and time in study activities.

**Aim 2 and 3:** *Healthcare providers* trained in intervention sites will implement PRO-ACT; and complete surveys and interviews. Additionally, providers in both intervention and control sites will be recruited to participate in surveys and interviews to assess the implementation of the intervention and determinants of implementation. They will receive reimbursement for their time completing surveys and participating in interviews.

*Adolescents and youth with HIV* will be recruited by study staff in the intervention and control clinics after the provider training and intervention optimization is complete during their routine clinic or refill visits. The clinic staff will introduce the study staff to the youth during triage who will then describe the aims of the study. Those interested in participating will undergo screening by the study staff before or after the clinic visit. All adolescents and youth ages 16-24 years in the participating clinics will be screened for psychological distress (symptoms for depression, anxiety or trauma) using recommended tools by the study staff. Approximately 300 participants in intervention clinics and 300 in control clinics with symptoms of psychological distress will be referred to the clinic staff for appropriate interventions (PRO-ACT in the intervention sites, standard of care in the control sites) and follow up. Phone contact information of the youth receiving the intervention and their caregivers or other trusted individuals that they identify will be collected by the study team. We will also collect and update their physical location information for tracking purposes where required. The adolescents will receive phone call reminders of their clinic visits and we will leverage the clinic mechanisms for home tracing/visits in the event that they are unreachable by phone. Youth that participate in the intervention will complete surveys and a sample will be invited for qualitative interviews to assess implementation outcomes and provide cost information. They will be reimbursed for transport to attend the sessions and for the time used in study activities.

**5f. Randomization procedures**

For the cluster randomized controlled trial, the 30 clinics will be randomized to intervention and control clinics. Clinics will be randomized using computer generated random numbers to either intervention or control status, using restricted randomization to balance for county. Randomization will be performed at UW by a biostatistician with access only to the clinic characteristics of interest. After randomization, the list of clinics and allocation arms will be given to the study team. Fifteen clinics will receive standard of care (screening and referral to available services) and 15 will receive the intervention (screening, PRO-ACT and referral to available services where applicable).

**5g. Masking procedures**

PRO-ACT will be administered at the clinic-level; therefore, it is not possible to blind participating clinics or study team members.

**5h. Participant Withdrawal**

Participants may withdraw from this study at any time. The Principal Investigator may also terminate a participant’s participation.

Reasons for Withdrawal

- Clinics may stop participation from the study if they face closure or major changes in HIV and mental health treatment policies or organizational structure that would negatively impact the conduct of the study.
- Providers may stop participation from the study if they face discomfort/ distress from the training or study procedure, or lack time to participate in the study procedures or if they face job termination or relocation from the study sites.
- Adolescents and youth with HIV may stop participation from the study if they face discomfort/ distress from the intervention, or lack time to participate in the study procedures, including completing surveys or participation in interviews.
- The Principal Investigator may terminate the continued participation of AYHIV or provider’s involvement in the study if they have a medical condition or are involved in a situation where continued participation in the study would not be in their best interest or if their behavior prevents the successful completion of study procedures.

Handling of participant withdrawals

- The study team will track and record the withdrawal of any clinic or participant during the study. The participants will be assured that their withdrawal from the study will not affect their employment or services received in the clinic. They will be thanked for their time in the study and not contacted further by the study team. Depending on the timing of the withdrawal, the Principal Investigator will make a decision on replacement of the clinic or participant.

**6. PRO-ACT INTERVENTION**

PRO-ACT is a transdiagnostic psychological intervention that includes 5 stand-alone modules with 5 modules to treat the most common mental health conditions (anxiety, depression, trauma-related anxiety). The intervention is based on the successful and effective Trauma-focused Cognitive Behavioral Therapy (TF-CBT) interventions, and will use similar intervention manuals with its lay provider-friendly setups (e.g., step sheets for each module) as the basis.

PRO-ACT includes: Psychoeducation, Relaxation, PrOblem solving, Activation, and Cognitive coping. A brief description for each module is provided below. Providers will be trained to select needed elements for an individual client, using vignette/case-based learning activities. All clients receive Psychoeducation; all other elements are selected based on client symptom presentation (Table 4).

| **Table 4: PRO-ACT modules** | |
| --- | --- |
| **Module** | **Description** |
| Psychoeducation | Psychoeducation- Information tailored to the particular client, regarding their mental health problem(s): depression, anxiety and/or traumatic experiences (PTSD)- These are interactive sessions that include normalization and validation. The provider shares what is known as common feelings and worries, thoughts and behaviors of children living with HIV. Psychoeducation provides an overview of PRO-ACT, provides hope, and begins the provider-client relationship. |
| Relaxation | Relaxation- Children/Youth are taught in an engaging way, tailored for developmental level/age, to recognize how stress is experienced in the body, learning to recognize and rate severity of stress/body tension and learning and different ways to relax the body to feel better. They are encouraged to make a table of events/feelings and practice at home, noting improvement for review with the provider. |
| Problem solving | Problem solving- Helps the young person identify and define a current problem that is causing anxiety or depression, that is in the young person’s control (e.g., they have some capacity to change or solve the problem). The counselor uses a problem-solving framework to brainstorm all possible solutions to the problem, determining the best possible solution to enact/try. In subsequent sessions, the provider and client can re-evaluate the problem and solution, repeating the cycle if need be. Provider helps the client work through internal (mood state) and external barriers to enacting solutions, and helps the client break solutions down into small steps, if needed. |
| Behavioral activation | Activation- For clients with depression who have decreased their own engagement in social activities or activities that bring them pleasure or meaning, the provider would apply behavioral activation. In the session, the counselor supports the client in identifying activities they can do in the week and rating impact on mood. The provider and youth explore possible/reachable activities that the youth can integrate in their day to day program. |
| Cognitive coping | In Cognitive Coping, clients learn that they can change their thinking to feel better (even if one cannot do something different).- Provider explains, with examples, how thoughts, feelings, and behavior are connected and how changing thought can change the way we feel and the way we behave- referred to as “thinking in a different way”. Demonstrates the Thought-Feeling-Behavior triangle using real examples. |

**7. STUDY PROCEDURES**

Study procedures for this study include the adaptation process with stakeholders, site selection and randomization, provider selection and training, a pre-trial PRO-ACT delivery optimization phase, randomized trial phase, and a post-trial phase to assess determinants of implementation.

**7a. PRO-ACT Adaptation procedures**

Stakeholder workshop

We will host a 2-day working group meeting with a purposively selected group of ~40 stakeholders described in section 5e to review the PRO-ACT intervention - The Intervention Adaptation Working Group (IAWG). The IAWG will review the modules (demonstrated via role play), training structure, and implementation approach. We will use a targeted agenda to determine adaptations regarding the service setting, target audience needs (AYHIV), and delivery mode for PRO-ACT;^121^ for example obtaining scenarios for cognitive coping and tailoring the implementation approach to have non-stigmatizing mental health screening methods. We will also obtain information to optimize PRO-ACT training, including where (in facility, out of facility) and when to host the training, as well as how to approach selection of non-specialists from facilities.

Stakeholders will weigh in their priorities and concerns regarding the intervention and its delivery, identify potential barriers to implementation of PRO-ACT and approaches to addressing them. We will engage stakeholders in barrier brainstorming followed by prioritization of barriers based on 4 factors: 1) criticality/importance, 2) ubiquity/pervasiveness, 3) chronicity/frequency and duration, and 4) equity (barriers disproportionately affecting specific groups of AYHIV like young key populations) on a 4-point Likert scale.^122^ Scores will be integrated across these four domains to create a single prioritized list. The stakeholders will then discuss potential solutions to the identified barrier that will be considered by the individual clinic level implementation teams in the pre-trial and trial phases. We will invite 10 IAWG members to join a core adaptation group (CAG), for ongoing engagement throughout the implementation of the study.

**7b. Site selection and entry**

The study team will present the ethical approvals for this study to the county health departments in Nairobi, Kisumu and Homa Bay counties. After their approval, the county teams will provide the list of public health facilities with HIV clinics and advise on the 40-50 facilities that should be selected based on the patient volume (>100 AYHIV), location and access (including urban/rural), and other studies being undertaken in the HIV clinic. The final list of 30 sites will be determined by the study team after visiting the facilities and confirming interest and ability of the sites to conduct the study. The selection of sites will ensure representation of lower versus higher volume sites (>200 AYHIV), facilities in rural versus urban areas in Kisumu and Homa Bay; and for Nairobi those in informal settlements versus other areas. The list will be sent to the study biostatistician for randomization - with the study arm allocation then communicated to the sites.

**7c. Provider training and supervision**

We will deploy up to five experienced PRO-ACT trainers from the pilot studies conducted in Kenya to conduct the standardized training for selected providers in the intervention facilities. The training and evaluation process (using the WHO ENACT tool) will be updated during the stakeholder engagement forums.

ENACT-15, adapted for EQUIP includes 15 competency items. They include non-verbal communication and active listening; verbal communication skills; explanation and promotion of confidentiality; rapport building and self-disclosure; exploration and normalization of feelings; demonstration of empathy, warmth and genuineness; assessment of harm to self, harm to others, harm from others and developing collaborative response plan; connection to social functioning and impact on life; exploration of client's and social support network's explanation for problem (causal and explanatory models; appropriate involvement of family members and relevant others; collaborative goal setting and addressing client’s expectations; promotion of realistic hope for change; incorporation of coping mechanisms & prior solutions; psychoeducation and use of local terminology; elicitation of feedback when providing advice, suggestions & recommendations. Each item is paired with a single-competency role play to support in appraising core competencies. Each appraisal item includes four levels of appraisal, level 1: any harmful behavior; level 2: any or no skills; level 3: all basic skills; level 4: any advanced skills.

We will utilize the adapted WHO ENACT competency tool through the WHO EQUIP platform to assess trainee competencies at three time points: pre-training, post-training and post-supervision, to target training and supervision needs for the trainees. The ENACT competencies will be assessed through virtual role-plays using trained standardized patient actors based on standardized vignettes developed by the research team and validated by stakeholders and providers during the pilot study and rated on the EQUIP digital platform by trained raters. Each training group will have a maximum of 10 providers. Training follows the Apprenticeship model: it is interactive with opportunities to practice all modules with peer and trainer feedback.^123^ We will conduct didactic training through online platforms like Zoom, and reserve the in-person training within clinics for role-playing. We will also stagger the training to take place during the less busy days and hours, and train smaller pods of providers per clinic so that service delivery is not interrupted or compromised. After the training, the providers will implement the intervention among one or two AYHIV in their facilities whom they screen and have mild to moderate symptoms of psychological distress (depressive symptoms, anxiety symptoms or trauma symptoms), supervised by the training psychologists over 3-6 months. The supervision will ensure there is fidelity in screening and delivery of PRO-ACT.

**7d. Continuous quality improvement (CQI)**

During the pre-trial period, (3-6 months after provider training), the study team will support intervention implementation through plan-do-study-act (PDSA) cycles aimed at adapting intervention delivery to increase intervention acceptability, feasibility and fidelity. Frontline providers in the adolescent HIV clinics will make small incremental adaptations to the implementation process including where, how, and when patients are reached (screened), development of job aids, and record-keeping (inpatient charts and electronic medical records). These changes – made in roughly two-weekly cycles (1 hour meeting and 2 weeks to study a small change) – are not geared towards fundamentally changing the intervention but optimizing its delivery and impact. They are also not expected to require additional equipment or human resources^124,125.^ The meetings are recorded and a survey completed during the meeting by the group, facilitated by study staff. The adaptations to the intervention delivery at clinic level through the CQI activities will be documented using the FRAME framework.

**7e. Cluster Randomized Controlled Trial (RCT)**

All AYHIV attending the 30 participating clinics will be screened for depressive, anxiety and trauma symptoms by study staff during their routine clinic visits. *This will differ from the required PHQ-9 or -2 mental health screening by clinic staff during routine visits.*^85^ Study staff will classify AYHIV based on severity of symptoms (mild/moderate/severe) as described above.

For inclusion in the cluster randomized trial, AYHIV eligibility criteria include: mild/moderate symptoms of depression, anxiety or trauma, age 16-24 years, willingness to consent to study procedures.

*Intervention sites:* AYHIV with mild/moderate symptoms of depression/anxiety/trauma will be offered PRO-ACT; all sessions will be delivered by trained providers in the clinic on an agreed schedule within 6 months. The study staff will conduct 3-monthly assessments for participating AYHIV via phone or in-person, and 6-monthly medical record abstraction for viral load results.

*Control sites:* AYHIV with mild/moderate symptoms will be offered referral (by the study staff) to available psychosocial support within the clinic as per standard of care practices. They will complete 3-monthly assessments (administered by study staff) and 6-montlhy medical record abstraction for viral load results.

*The standard of care:* Kenyan guidelines for the provision of HIV services and adolescent friendly services recommend screening of all AYHIV during their routine clinic visits using the PHQ2/9. While psychotherapy is recommended for those with elevated depressive symptoms, the provision of this service is dependent on whether there is a provider in the facility with training to offer it. ART adherence counseling is however offered by nurse counselors, which includes aspects of motivational interviewing and problem solving techniques. HIV clinic-mental health specialist interfaces though recommended are not developed in majority of clinics.^109,120^

In both intervention and control sites, AYHIV who have severe symptoms or who have suicidal ideation will be referred for specialized care. In Nairobi Youth will be referred to KNH youth Clinic, Mathari hospital CHild and Adolescent clinic or Ngaira Youth clinic- Nairobi County. In Kisumu and Homa Bay youth will be referred to the nearest level 4 or 5 county hospital with psychiatric services.

Table 1 above summarizes the study procedures to assess effectiveness of PRO-ACT

**7f. Assessing implementation of the intervention**

To assess the implementation of the intervention, the study will conduct the following procedures (summarized in Table 2).

Patient records will provide data on mental health screening using PHQ2/9 for AYHIV in 30 clinics, 6-months prior to the start of the study, and up to 24 months after site entry. These data will inform assessments of **reach** (~2400 records) and **maintenance** (~1200 records).

A checklist will be developed and will accompany patient charts (~2400 charts) and completed by clinic staff to assess implementation/fidelity of mental health screening. In the intervention sites (~1200 charts), the checklist will also be used to assess **implementation/fidelity** focusing on adherence to PRO-ACT by providers for ~300 AYHIV. The checklist in the intervention sites will have an observer component to be completed by trainers (~100 records). The observer form allows rating of both adherence and of competence.

Study administrative records for the 30 sites will be used to track which study sites are trained and elect to deliver PRO-ACT, informing assessment of **adoption**.

A self-administered survey with providers in the intervention sites (~6 per site) will assess **acceptability** of PRO-ACT after training using the Acceptability of Intervention Measure (AIM) score and satisfaction with the training.

For trained providers in intervention sites (~6 per site), their **competence** pre-training, immediately after training and after completing supervision will be assessed using the WHO, EQUIP-developed ENACT provider competency assessment tool, within the virtual EQUIP platform. The providers will role play sessions with standardized patient actors trained using standard vignettes, which will be rated by study-trained raters within the study (following standardized/established EQUIP procedures as used in the Nairobi pilot of PRO-ACT).

Adolescent **satisfaction** with mental health services offered in clinics and cost of care will be assessed using a survey. All AYHIV will complete the survey at baseline and month 12.

*Interviews & focus groups with providers to assess determinants of implementation*: We will conduct up to 6 in-depth interviews (IDIs) and 1 focus group discussion (FGD) at each of the 30 sites at end line to assess determinants of implementation of the intervention PRO-ACT (intervention sites) and reach of screening (all sites). Each FGD will consist of 7-10 participants. For in-depth interviews, we will recruit 2 lower cadre staff members (e.g. peer counselor), 2 middle cadre staff members (e.g. nurse or clinical officer), and 2 managers (e.g. medical superintendent) from each facility. Interviews will complement the FGDs and are included to collect potentially sensitive information that staff members might be hesitant to share in FGD settings with superiors or subordinates. FGDs and IDIs will be conducted by an experienced interviewer/facilitator accompanied by a note-taker (FGDs only), audio-recorded, translated (as necessary), and transcribed. While we expect that most providers will feel comfortable with discussions in English, we will select an interviewer who is fluent in English, Swahili and Dholuo, reflecting the dominant languages spoken in our study area. Information obtained from control site providers will be compared and contrasted to those from the implementation site providers to assess the critical role of the different determinants of reach of screening.

| **Table 5: Roles for study staff and clinic staff in the PRO-ACT trial** | |
| --- | --- |
| **Clinic staff** | **Study staff** |
| Introduce AYHIV to study staff to conduct recruitment and mental health screening | Conduct recruitment and mental health screening of AYHIV |
| Conduct routine mental health screening as per guidelines - standard of care | Refer AYHIV with mild to moderate symptoms of psychological distress to clinic staff (intervention clinic: for PRO-ACT intervention; control clinic: for standard of care) |
| Trained staff at intervention sites administer PRO-ACT to AYHIV with mild to moderate symptoms of psychological distress | Refer AYHIV with severe symptoms of psychological distress to clinic staff for further referral to mental health specialists and follow up their care |
| Refer AYHIV with severe symptoms of psychological distress to a mental health specialist, and follow up with the | Conduct follow up surveys with AYHIV in the trial (both intervention and control clinics) |

**8. STUDY SCHEDULE**

The study will adapt the PRO-ACT intervention for the AYHIV care settings in year 1; conduct site selection and entry in year 1; complete provider training and supervision and pre-trial intervention optimization in year 1 and 2; screen, enroll and follow up AYHIV participants in the trial in year 2, 3 and 4; collect effectiveness, implementation and cost data in year 2, 3 and 4; complete analyses and dissemination including manuscript writing in year 4 and 5; and write a follow up grant in year 5. Table 5 shows a schedule of activities.

| **Table 6: Schedule of activities** | | | | | |
| --- | --- | --- | --- | --- | --- |
| **Activity** | **Y1** | **Y2** | **Y3** | **Y4** | **Y5** |
| Adaptation of PRO-ACT through a stakeholder-led process |  |  |  |  |  |
| Site selection and entry |  |  |  |  |  |
| Provider training and pre-trial intervention optimization |  |  |  |  |  |
| Participant recruitment and enrolment and follow up |  |  |  |  |  |
| Participant follow up and exit |  |  |  |  |  |
| Data collection for effectiveness, implementation and economic evaluation |  |  |  |  |  |
| Analyses |  |  |  |  |  |
| Local and international dissemination and manuscript writing |  |  |  |  |  |
| Follow up proposal |  |  |  |  |  |

**9. ASSESSMENT OF SAFETY**

The PRO-ACT study is a randomized trial of a psychological intervention. Being a behavioral intervention, we anticipate no risk of serious harm to participating providers or AYHIV.

**9a. Psychological and emotional distress:** Some AYHIV may find assessments of their mental health status stressful. All reasonable efforts will be made to minimize the risks of loss of privacy or psychological stress experienced in the interview process. Research personnel will be properly trained and educated. All assessments will be conducted in private. Any AYHIV who experiences prolonged distress during the assessment process will have the opportunity to skip questions that are distressing or stop the interview. Clinic and study staff will be trained in basic counselling to provide immediate support as needed.

**9b. Referral for specialized care:** AYHIV who are scored severe on the assessment tools for depression, anxiety or PTSD or where active suicidality is elicited will require immediate referral and follow-up or peer accompaniment to ensure the youth gets to the referral facility . The PHQ-9 has one screening question for suicidality and if positive a simple 4 question Suicidal Bheaviour Questionnaire will be used to determine active suicidality. The study team will build referral pathways specific for every clinic based on existing resources to ensure AYHIV are referred appropriately. Specifically, at the start of each training phase, the research training team and a designated county mental health provider (e.g. a psychologist) will review procedures at each participating intervention site for referral of AYHIV with severe distress, including contacting a county mental health specialist for a) telephone consultation and b) referral to designated health care facilities for evaluation and further management. In Nairobi County, Kenyatta National Teaching and Referral Hospital (KNH) and Mathari Teaching and Referral Hospital both offer outpatient child and adolescent psychiatric clinics. The KNH youth clinic is situated at the free youth friendly clinic, which offers free walk-in services to youth from 12 years to 25 years. Additionally Nairobi County has recently started offering a child and adolescent mental health services with psychiatrists as well as psychologists at Ngaira Health Centre. The study team includes investigators who are consultant psychiatrists and psychologists attached to both teaching hospitals and will be able to facilitate referrals to both institutions in collaboration with the Nairobi County psychologists. In Kisumu and Homa Bay, the study team will collaborate with the County health department to establish a clear referral procedure to Hospitals with a mental health specialist including Jaramogi Oginga Odinga Teaching and Referral Hospital and Homa Bay County Hospital.

**9c. Other referrals:** During treatment sessions, it is also possible that providers will learn about issues such as child sexual and physical abuse in or outside the home or about parent/guardian depression or other serious mental health problems. Sexual abuse in Kenya is a notifiable offense. We will develop an emergency protocol that is within the framework of Kenyan legal requirements and organize for the survivors to be seen at the KNH Gender Based Recovery Centre, which is a “one stop” service in Nairobi County. Similar procedures will be outlined at the start of the study, in collaboration with clinic and county managers, for referrals in Homabay.

**9d. Quality of care and provider competence:** The study procedures do not interfere with routine care. The study will train providers to use a treatment approach with which they may be unfamiliar (psychological therapy). We will take precautions to ensure that the trained providers are able to safely deliver care to AYHIV. Providers who receive training in the PRO-ACT intervention will be clinical providers affiliated with public HIV clinics in 2 counties in Kenya. During the PRO-ACT training, trainers will closely observe trainees for their grasp of the material, ability to respond to changing situations during role-plays, and responsiveness to direction. In this study, we will use the ENACT competency assessment tool to assess provider competency; scores below the designated threshold will disqualify a provider from delivering the intervention. We will discuss each provider’s competency within the Kenya and UW research trainers and intervention experts.

Additional safety measures will include i) the training of the trainers on child protection and safety (i.e. all trainees will review and sign Child Protection or Vulnerable Adults policies, which provide them high-level guidelines for maintaining the safety of vulnerable groups); ii) articulation of a clear engagement and referral procedure when additional care is needed; and iii) as per University of Nairobi Research Ethics Committee requirements, depending on age, adolescents and their parent /guardian will sign an assent or consent form respectively.

**9e. Safety of providers:** We anticipate minimal risk to providers during the study; however, we recognize that they will be asked to role-play and listen to and respond to potentially distressing scenarios when delivering care. Sometimes people can experience emotional distress while engaging in such role-playing or while engaging in care delivery. We will advise all study participants of this potential risk. Counselling staff will be available for support in the event that any participant experiences distress during the role plays. Providers who will administer the intervention to AYHIV patients during the study will receive at least bi-weekly supervision, which provides an opportunity to express distress and to receive support. Participants will be advised that they are free to stop role playing or delivering the PRO-ACT intervention should they feel distressed. Each training session batch will integrate measures for self-care for all training/trainee participants.

**9f. Specification of Safety Parameters**

The Office for Human Research Protections (OHRP) defines risks that fall in this study as ‘unanticipated problems’.. Unanticipated problems are defined as a problem or event that meets all of the following criteria:

- Unexpected
- Related or possibly related to participation in the research
- Suggests that the research places (or could have placed) participants or others at a greater risk of harm than was previously known or recognized. This includes physical, psychological, economic or social harm

We will periodically monitor all study sites for unanticipated problems through observation and feedback from providers and patients, and record any unanticipated problems in a study database.

**9g. Reporting of unanticipated problems and other events**

In compliance with USA federal regulations and UW policy, the Principal Investigator will notify the Kenyatta National Hospital/ University of Nairobi Ethics and Research Committee (KNH/UoN ERC) of any unanticipated problems within 10 business days. Any breach or possible breach of confidentiality of any participants in this study will be reported to the KNH/UoN ERC within 24 hours.

**10.** **STUDY OVERSIGHT**

An External Advisory Board (EAB) will be selected to provide oversight of this study. Members of the EAB will be selected by the Principal Investigator, with their names and details forwarded to the funding organization - The National Institute of Mental Health (NIMH). The members will be from diverse backgrounds and professional competencies. Their diversity will maximize the relevance and impact of project activities through ongoing assessment of the research's progress and discussion of future research goals, aims, and ideas. EAB members will include senior NIH-funded researchers in Kenya and in the broader HIV and global mental health communities. Other members from Kenya will be mental health and HIV care providers, a representative of an AYHIV-serving community organization, youth leaders, and county-level health officials. We will develop a governance structure in collaboration with the 10-12 recruited members. We will convene two full advisory meetings per year and will inform the EAB of activities quarterly, engaging members with specific areas of expertise as needed according to study activities. EAB meeting reports will be incorporated into annual progress reports to NIMH.

**11.** **DATA COLLECTION**

The data collection tools to be utilized in this study can be found in the Appendices.

**11a. Data collection tools**

1. *Stakeholders workshop audio-recordings and notes:* The stakeholder workshop sessions will be audio-recorded and transcribed by a study team member. Notes will also be taken by one of the group members. Participants will provide written consent for participation in the audio-recorded sessions. No specific measures will be taken to conceal the identity of any participant. Measures to ensure that access to the recordings and notes is only by study staff will be taken, such as storing them in a lockable cabinet, and only making them accessible to authorized study team members doing transcription or analysis of the content of the recordings.
2. *Surveys:*

- *Stakeholders socio-demographic survey:* Participants in the stakeholders workshop will complete a brief socio-demographic survey.
- *Facility survey:* The head of each facility/ HIV clinic or their designated employee will complete a survey to document the various services offered in their facility/clinic including mental health services, barriers and facilitators to integration of services to HIV services for AYHIV, and the organizational readiness for change.
- *Provider sociodemographic and satisfaction survey*: Providers in the selected intervention and control sites will complete a survey on sociodemographic information, and satisfaction with how they offer services at baseline and at study exit.
- *Provider training satisfaction and competence survey:* Providers in the intervention sites participating in the PRO-ACT training will complete a survey at 3 timepoints - pre-training, post-training and post-supervision to assess competence, satisfaction with the training and supervision
- *Intervention implementation provider survey:* Providers in intervention sites implementing the PRO-ACT intervention will complete a survey at month 3 and month 12 to assess acceptability of PRO-ACT, implementation/ fidelity and maintenance.
- *Qualitative interview socio-demographic survey:* Providers in intervention and control sites participating in qualitative interviews will complete a brief form collecting socio-demographic data

- *Adolescent and youth screening, satisfaction and cost survey:* All AYHIV in participating sites ages 16-24 years will complete a survey collecting information on sociodemographics and screening for common mental health conditions, and other social and HIV related information, satisfaction with services, and cost information at baseline and at month 12.
- *Adolescent and youth intervention follow up survey:* All AYHIV with mild to moderate symptoms of psychological distress enrolled into the trial will complete a survey collecting information on sociodemographics and screening for common mental health conditions, and other social and HIV related information, and cost information every 3 months for 12 months.

1. *Program records:*

- *Program cost data:* Program data (e.g. provider salaries) will be collected using an Excel spreadsheet at baseline and updated as needed throughout and immediately after the trial.
- *Adolescent routine clinic records:* De-identified adolescent clinic records from the electronic medical records (EMR) system and physical charts will be abstracted at baseline, and within 18 months after starting the intervention. Examples of data will be visit dates, enrollment status (newly enrolled or existing), date of birth/age, sex, viral load and WHO clinical staging, pharmacy records, and referrals to allied services.

1. *Qualitative interview guides:* In-depth interview and Focus Group Discussion guides will include questions adapted from the Consolidated Framework for Implementation Research (CFIR) question bank. The topics covered in each type of data collection will not differ, as we are interested in understanding group norms about each topic (achieved with FGDs) as well as noting whether there are minority opinions withheld during group discussion (identified with IDIs). This knowledge will be useful in understanding heterogeneity in trial outcomes and determinants of implementation.
2. *Clinical workflow maps:* During the first 2-3 CQI meetings, the clinic and study team will map out how services are delivered for AYHIV in the HIV clinics.
3. *Time in Motion studies:* Time and motion studies will be conducted to estimate provider time offering services to AYHIV (~6 providers per facility), which will be used to reliably apportion effort to the providers implementing the activities who divide time and effort among multiple roles. This will be through observation by study staff following AYHIV receiving care from the selected providers.
4. *Checklists*

- *Adolescents and young adults recruitment log:* The study staff will keep a record of each AYHIV that visited the facility and note whether they were recruited for screening, reasons for declining or not completing screening, and whether they were enrolled for the trial.
- *PRO-ACT modules checklist:* Providers in intervention sites will keep a record of the modules they start and complete in each visit with each AYHIV in PRO-ACT.
- *PRO-ACT fidelity checklist:* Providers and study staff will complete checklists to assess fidelity of the implementation of the intervention.

**11b. Data Management Responsibilities**

Data management will be overseen by a dedicated data team, leading research assistants in entry, management, and monitoring of study data, in accordance with standard operating procedures. Under the guidance of the study PI, the team will also be responsible for data cleaning, and interim analyses.

**11c. Data Capture and Storage Methods**

- All *primary collected and abstracted data* will be entered into a RedCap study database for storage and management. Data collection will be done using phones/ tablets using tools developed through the mobile RedCap. All Study Staff will be trained in data protection and privacy. RedCap databases are encrypted and enabled with 2-step verification to restrict user access. The link between study identifiers and patient IDs will be kept under lock and key. The link between patient identifiers and Study ID codes will be retained until completion of the study.
- The methods of *EMR data* abstraction will follow a data use agreement between the study team and NASCOP/study sites. We will provide a list of variables and records for a data administrator to abstract the records. We will receive data in a secure, password protected format (e.g. USB or cloud server). These data will be stored in a password-protected database accessible only to authorized members of the study team.
- *Audio-recordings* will be stored on Canvas, a secured learning management system (LMS) utilized by Kenyatta National Hospital and the University of Washington. The LMS uses industry standard security and will be password protected.

**11d. Data Custody and Retrieval Procedures**

The Principal Investigator and authorized study staff will be the custodian of all the study data. Data retrieval procedures will be similar for all types of data in this study. Authorized study staff members will download the datasets from the secure servers (i.e. surveys and audio recordings) for routine quality checking and analyses. All downloaded data will be maintained on a secured, password-protected study computer.

**11e. Study Records Retention**

Retention of study records will comply with the Kenyan data laws, and the US Federal and University of Washington requirements where applicable:

(<http://f2.washington.edu/fm/recmgt/retentionschedules/gs/general/uwgsResearch#Research>).

All study data and link between participant identifiers and study ID codes in the anonymized data will be retained for 6 years following completion of the study. Audio-visual data will be destroyed after 6 years, unless a waiver has been signed. Participant consents will be retained for 6 years after the end of the study. The link between participant identifiers and study IDs will be kept under lock and key. After this time, links to identifiable data will be destroyed.

**12.** **STATISTICAL CONSIDERATIONS**

**12a. Sample Size Considerations**

**Aim 1:** *Stakeholders’ workshop:* We estimate that 40 purposively selected participants for the stakeholders’ workshop will be sufficient to provide diverse information leading to the contextual adaptation of the intervention and the implementation process. Our previous work to develop interventions in Kenya has included 30-40 stakeholders which has been sufficient to reach saturation in perspectives.

**Aim 2**: *Cluster randomized controlled trial:* From previous work, we anticipate about 25% of adolescents and young adults in each clinic will have mild to moderate mental health scores and enroll in the study. We anticipate enrolling 10 to 20 participants from each of 30 sites. Table 1 contains conservative estimates of statistical power to detect a range of smaller (Cohen’s *d* = 0.2, 0.3, and 0.4) effects under different study scenarios for an alpha value of 0.05. They are conservative estimates because they assume little predictive power of covariates in the random coefficients models (i.e., R^2^ values of 0.05 for the individual level and 0.01 at the site level).

| **Table 5: Conservative power estimates** | | | | | | | | | | |
| --- | --- | --- | --- | --- | --- | --- | --- | --- | --- | --- |
|  | Intraclass correlation values | | | | | | | | | |
| Number of participants per site | 0.01 | | | 0.03 | | | 0.05 | | | |
| ‍ | Effect size | | | Effect size | | | Effect size | | | |
|  | **0.2** | **0.3** | **0.4** | **0.2** | **0.3** | **0.4** | **0.2** | **0.3** | **0.4** |  |
| 10 | 0.37 | 0.69 | *0.91* | 0.33 | 0.62 | *0.86* | 0.29 | 0.56 | *0.81* |  |
| 20 | 0.6 | *0.91* | *0.99* | 0.48 | *0.82* | *0.97* | 0.40 | 0.73 | 0.93 |  |

**Aim 2:** *Implementation outcomes:* Table 6 contains the minimum detectable effects for binary and continuous implementation science outcomes under the design described above. These power estimates are for a power of 0.8, alpha of 0.05, and R^2^ = 0.05.

| **Table 6: Minimum detectable effects for implementation science outcomes** | | | | | | | |
| --- | --- | --- | --- | --- | --- | --- | --- |
| **Binary outcomes** | | | | | | | |
| ‍ | Minimum proportion difference from 0.8 | | |  | Minimum proportion difference from 0.4 | | |
| ‍ | Intraclass correlation values | | |  | Intraclass correlation values | | |
|  | 0.01 | 0.03 | 0.05 |  | 0.01 | 0.03 | 0.05 |
| Reach (~80 per site) | 0.73 | 0.71 | 0.69 |  | 0.32 | 0.30 | 0.28 |
| Maintenance (~40 per site) | 0.72 | 0.69 | 0.67 |  | 0.31 | 0.28 | 0.27 |
| Provider (~ 6 per site) | 0.60 | 0.59 | 0.58 |  | 0.20 | 0.19* | 0.19* |
| ‍Site (30) | 0.34** |  |  |  | 0.02** |  |  |
| **Continuous outcomes** | | | | | | | |
| ‍ | Minimal detectable Cohen’s d | | |  |  | | |
| Patient (~ 80 per site) | 0.16 | 0.22 | 0.26 |  |  |  |  |
| ‍Provider (~ 6 per site) | 0.43 | 0.46 | 0.48 |  |  |  |  |
| ‍Site (30) | 1.03** |  |  |  |  |  |  |

**Aim 2:** *Determinants of implementation - qualitative study:* We will conduct up to 6 in-depth interviews (IDIs) and 1 focus group discussion (FGD) at each of the 30 sites with providers to assess determinants of implementation of the intervention PRO-ACT (intervention sites) and screening (all sites). Each FGD will consist of 7-10 participants, sufficient to generate conversation without being too large to become less manageable . For in-depth interviews, we will recruit 2 lower cadre staff members (e.g. peer counselor), 2 middle cadre staff members (e.g. nurse or clinical officer), and 2 managers (e.g. medical superintendent) from each facility. Based on the qualitative evaluation from past hybrid trials and the literature, we expect that 6 IDIs per site and 1 FGD per site will be sufficient to reach thematic saturation.

**Aim 3:** *Economic evaluation:* The program cost data will be extracted from project expenditure records, and collected through serial structured interviews with heads of clinics or their representatives in facilities (30). Time and motion studies will be conducted to estimate provider time offering services to AYHIV (~6 providers per facility), which will be used to reliably apportion effort to the providers implementing the activities who divide time and effort among multiple roles. The longitudinal patient survey will collect costs of seeking care from the ~600 AYHIV participating in the trial.

**12b. Planned interim analyses (if applicable)**

Analyses of the data from the stakeholders workshop, and the CQI process will inform adaptation and refinement of the PRO-ACT intervention and its delivery. No other interim analyses are planned or stopping rules.

**12c. Overview of analysis plan**

***Analysis of the effectiveness of PRO-ACT***

***Primary analyses:*** Primary analysis of the effectiveness of PRO-ACT will be conducted at 6- and 12-month follow-up. Mental health data for the primary analyses including assessments for depressive, anxiety and trauma symptoms will be collected at baseline, and follow up at months 3, 6, 9, and 12 for AYHIV with mild to moderate symptoms in intervention and control clinics. Assuming that most or all participants have completed the intervention by then, the 6-month analysis provides an estimate of the near-term treatment effect, while the 12-month analysis examines treatment effect sustainability. Both 6- and 12-month analyses will employ random coefficients models to account for clustered data structure, and compare outcome means separately (depression, anxiety, trauma) between the treatment and control groups. These models will include baseline symptom scores as predictors, as well as county, demographics, school (boarding vs. day), and clinic type (youth specific vs. integrated)as covariates. The impact of these covariates as treatment moderators will be examined. Model assumptions will be checked, including normality, linearity of effects, additional predictor interactions, and power-terms, including among baseline mental health scores (e.g., anxiety x depression interaction). We will then add provider competency to the final outcome models to examine its role as a mediator of the treatment effect.

***Secondary analyses:*** Viral load data will be abstracted from clinic records at baseline, month 6 and month 12. Viral suppression analyses at 6- and 12-month follow-ups will use similar clustered data models for the group randomized structure and strategies, as described for the analysis of the mental health outcomes, however for a binary response (i.e., relative risk regression). The same covariates and interactions will be included in the model with the treatment/control indicator variable. Model assumptions of effects and additional interactions will also be checked.

***Analyses for implementation outcomes and determinants of implementation***

- *Quantitative analysis of implementation outcomes* will follow a similar strategy to the mental health outcomes described above. Record and provider level analyses will require random coefficient models to accommodate clustering within site. The treatment/control indicator, covariates, and important interactions will be examined, as will model assumptions for each implementation outcome.
- *Qualitative data:* Two primary coders will independently code the transcripts from the IDIs and FGDs, coordinating their coding to create a comprehensive codebook based in the Consolidated Framework for Implementation Research (CFIR) codebook, and conducting a thematic analysis. Should new constructs emerge during our predominantly deductive coding, we will include them inductively. Notes and transcripts from the stakeholders workshop will be analyzed as qualitative data.

***Economic evaluation of PRO-ACT***

*The unit costs* (relevant program and patient costs divided by outputs) will be calculated using a health system perspective as well as patients’ perspective. The costing approach will emphasize resources utilized, rather than out-of-pocket costs by including resources like donations or transfer payments as required. Costs for capital items will be amortized on a straight-line basis over their expected useful life, and assuming no salvage value. Facility space required for the intervention will be valued at the market rental rate. Outputs (denominator of the unit cost) include the numbers of patients receiving each type of study-supported services. We will assess the variation in unit costs across the study sites and identify the major determinants of that variation. We will compute the changes in unit cost over time as programs potentially achieve greater scale and administrative efficiency. We will also calculate the cost per additional AYHIV receiving mental health services.

*Estimating incremental cost-effectiveness:* This is the net added cost per case of depressive/anxiety/trauma symptoms resolved (health outcome). The numerator will reflect differences in the costs for the intervention arm and control arm. The denominator will represent the difference in the health outcome due to the clinical benefits of the intervention. The ratio is the incremental cost-effectiveness ratio (ICER) in dollars per health outcome.

**13. ETHICS/PROTECTION OF STUDY PARTICIPANTS**

**13a. Informed consent:**

All participants who will be engaged directly in study procedures will be informed of the study by a nurse or another member of the research team. Informed consent will be required for participation as follows:

a. Baseline screening for all AYHIV age 16-24 years: oral consent for mental health screening

b. To participate in the trial, AYHIV with mild to moderate symptoms ages 16-17 years: written informed consent for participation will be sought from the parent/ caregiver and written assent from the adolescent.

c. To participate in the trial, AYHIV with mild to moderate symptoms ages 16-17 years **who present for care unaccompanied:** We will request a waiver of parental consent from regulatory bodies. From previous studies, youth age 14+ present to clinic alone. Excluding this population or requiring parental consent to participate reduces generalizability. Youth in this category will provide written informed assent to participate.

d. To participate in the trial, AYHIV ≥18 with mild to moderate symptoms will provide their own written informed consent for participation

e. Providers ≥18 years will provide written informed consent for participation in the study, including training, in the trial, and qualitative interviews.

f. Stakeholders (including policymakers, providers, caregivers of AYHIV, AYHIV) ≥18 years will provide written informed consent for participation. We will request a waiver of parental consent from regulatory bodies for AYHIV <18 years who will be nominated to participate in the stakeholders workshop.

*Consent Details:* A member of the research team will review the informed consent document, which states the purpose of the study and the procedures and content of all interviews. For participants in the clinical trial, the consent form will also describe the randomization procedure. The consent forms will convey the principles of respect for persons and will be developed with adequate details regarding study procedures and risks in a language and level that is understood by the participants. The consent will be translated into Kiswahili and other local language (e.g., Dholuo) as required to ensure that participants fully understand it. The voluntary choice to participate, decline or withdraw from the study without consequences, such as loss of services, will be clearly communicated and there will be no coercion or inducement to participate. After going through the consent form with the participant, the study staff will answer questions from the participant, then ask targeted questions to evaluate their understanding of the study; the purpose of the study, procedures, risks and benefits. The open-ended questions will be easy to respond to and in the language of choice of the participant, for example: what have you understood to be the reason why we are conducting this study, why are you being involved in this study, what is expected of you as a participant in the study, what are the possible risks for your involvement in the study, what are the possible benefits for your involvement in the study.

**13b. Disclosure of HIV status:** All enrolled AYHIV will be HIV-positive and aware of their HIV status. To avoid inadvertent HIV disclosure, the study team will work with clinic counsellors to identify AYHIV who are aware of their HIV status. The study team will confirm by asking the AYHIV questions like “Why do you take your medicine?” Only those who respond by saying they have HIV will be enrolled. All study team members will receive training on handling human subjects in research and good clinical practice and will make effort to ensure that a participant’s HIV status is not inadvertently disclosed through the study procedures and study data.

**13c. Confidentiality:** To maintain confidentiality data will be collected and stored in strict compliance with principles of good clinical practice. All audio records from FGDs and IDIs will be stored in secure devices with encryption and password protection restricted access only to authorized study personnel. Data abstraction will be guided by standard operating procedures to ensure that there is no leakage, and all databases will be secured. Study teams will be trained on existing data safety measures available at participating clinics to ensure they maintain confidentiality of participant chart records during record abstraction. Physical documents will be locked in secure cabinets with keys accessed by designated staff. All study personnel will be required to complete refresher training on human subjects and good clinical practice.

**13d. IRB approval and oversight:** Before initiation of the study the protocol will be reviewed by the institutional review board at the University of Nairobi and Kenyatta National Hospital (UON-KNH ERC) and the University of Washington. The consent documents, questionnaires and other study documents will be submitted to the ERC for review and approval before they are used. Participants will be given contact to the representative of the Ethics board in the event that they need to contact the Ethics bodies.

**13e. Reimbursements:** All study participants (including youth) will receive reimbursement for their time and/or travel at rates determined by the local ethical board. Currently, this is approximately KES 600 for completing study procedures including surveys and interviews below 1 hour, and KES 1000 for procedures requiring more than an hour, and not part of their usual clinical care or duties. In cases where a minor is accompanied by their parent, only the minor will be reimbursed and the parent will be informed of the reimbursement. The study will follow reimbursement guidelines by the local ethics review committee at KNH that periodically reviews them to cater for increases in the cost of living.

**13f. Procedures to minimize psychological risks:** *Note that in section 9 (Assessment of safety) we detail some potential risks to the AYHIV and providers and their mitigation.* To minimize emotional distress counselling support will be available for any participant who gets unduly distressed. Study personnel administering questionnaires on sensitive subjects will assure participants that they can skip questions that they find too intrusive with no consequence to them.

**13g. Additional protection for children:** The study meets CFR 46.406 “Research involving greater than minimal risk and no prospect of direct benefit to individual participants, but likely to yield generalizable knowledge about the participant's disorder or condition.”

(a) The risk represents a minor increase over minimal risk;

(b) The psychological intervention presents experiences to participants that are reasonably commensurate with those inherent in their actual or expected medical, dental, psychological, social, or educational situations;

(c) The study is likely to yield generalizable knowledge about the participants' disorder or condition which is of vital importance for the understanding or amelioration of the participants' disorder or condition; and

(d) Adequate provisions are made for soliciting assent of the children and permission of their parents or guardians, as set forth in §46.408.

**13h. Potential Benefits:** The proposed study has potential to integrate mental health to existing care services for AYHIV. Mental health challenges are prevalent among AYHIV and are a major driver for non-adherence, non-viral suppression and loss to follow-up. The study will provide real world information on integration practice as well as critical effectiveness data of an existing mental health intervention. This information has the potential to directly benefit individual AYHIV through improved mental health. The information could also benefit other AYHIV in similar settings on how to integrate mental health services into existing HIV clinic services.

**13i. Incidental findings**: All AYHIV who are identified to have psychosocial distress or other challenges will be linked to appropriate services.

**13j. Alternative treatments or procedures***:* Participants will not be disadvantaged in any way by declining participation in this study in regards to their ability to receive their usual care in the clinic, or to be involved in other ongoing or future studies at the facility.

**Budget summary (2023-2028)**

| **CATEGORY** | **COST (USD)** |
| --- | --- |
| Salaries and benefits | 1,372,553.05 |
| Consultants | 89,600.00 |
| Intervention adaptation and optimization | 65,320.00 |
| Site entry, execution and evaluation of intervention | 83,792.00 |
| Participant reimbursement | 35,000.00 |
| Subcontracts | 700,750.94 |
| Travel | 101,174.00 |
| Supplies | 51,805.00 |
| TOTAL DIRECT COSTS | 2,499,994.99 |
| TOTAL MTDC INDIRECT COSTS | 153,939.53 |
| **TOTAL COSTS** | **2,653,934.52** |

**References**

1. World Health Organisation. World mental health report: Transforming mental health for all report. 2022. Cited May 8, 2023. Available from<https://www.who.int/publications/i/item/9789240049338>

2. Galderisi S, Heinz A, Kastrup M, Beezhold J, Sartorius N. Toward a new definition of mental health. World psychiatry. 2015 Jun;14(2):231. Available from<https://ueaeprints.uea.ac.uk/id/eprint/65104/1/Final_Galderisi_Mental_Health_Definition.pdf>

3. Institute of Health Metrics and Evaluation. Global Health Data Exchange (GHDx), (<https://vizhub.healthdata.org/gbd-results/>, accessed 14 May 2022).

4.  [Mental Health and COVID-19: Early evidence of the pandemic’s impact](https://www.who.int/publications-detail-redirect/WHO-2019-nCoV-Sci_Brief-Mental_health-2022.1). Geneva: World Health Organization; 2022.

5. Silva SA, Silva SU, Ronca DB, Gonçalves VSS, Dutra ES, Carvalho KMB. Common mental disorders prevalence in adolescents: A systematic review and meta-analyses. PLoS One. 2020 Apr 23;15(4):e0232007. doi: 10.1371/journal.pone.0232007. PMID: 32324835; PMCID: PMC7179924.

6. World Bank Group. The 2017 Atlas of Sustainable Development Goals. 2017; Available from: <https://blogs.worldbank.org/opendata/2017-atlas-sustainable-development-goals-new-visual-guide-data-and-development>.

7. Marmot M, Friel S, Bell R, Houweling TA, Taylor S, Health CoSDo. Closing the gap in a generation: health equity through action on the social determinants of health. Lancet. 2008;372(9650):1661–9.

8. Scheidgen S, Klingler C, Bertram T, Rogowski WH, Marckmann G. What is personalized medicine: sharpening a vague term based on a systematic literature review. BMC Med Ethics. 2013;14:55.

9. Shah J, Scott J. Concepts and misconceptions regarding clinical staging models. J Psychiatry Neurosci. 2016;41(6):E83–4.

10. Allen D, Gillen E, Rixson L. The effectiveness of integrated care pathways for adults and children in health care settings: a systematic review. JBI Libr Syst Rev. 2009;7(3):80–129.

11. Stevens M. The costs and benefits of early interventions for vulnerable children and families to promote social and emotional wellbeing: economics briefing. London: National Institute for Health and Care Excellence; 2011.

12. Kessler RC, Berglund P, Demler O, Jin R, Merikangas KR, Walters EE. Lifetime prevalence and age-of-onset distributions of DSM-IV disorders in the National Comorbidity Survey Replication. Arch Gen Psychiatry. 2005;62(6):593–602.

13. World Health Organization. Guidelines on mental health promotive and preventive interventions for adolescents, helping adolescents thrive. 2020. Available from<https://apps.who.int/iris/bitstream/handle/10665/336864/9789240011854-eng.pdf>

14. Government of Kenya (GoK). The Constitution of Kenya 2010. 2010. Nairobi: Government Printer. Available from<http://kenyalaw.org:8181/exist/kenyalex/actview.xql?actid=CAP.%20248#:~:text=that%20interventions%20for%20the%20care,27%20of%202022%2C%20s>.

15. Ministry of Health (GoK). National Guidelines for Provision of Adolescent Youth-Friendly Services (YFS) in Kenya. 2005. MoH division of Reproductive health

16. Ministry of Health (GoK). National Guidelines for Provision of Adolescent Youth Friendly Services in Kenya. 2016. MoH Division of Family Health

17. Ministry of Health (GoK). A Comprehensive Guid on Mental Health and Psychosocial Support during the COVID-19 pandemic. 2020. MoH Kenya

18. Ministry of Health (GoK). Mental Health Policy. 2015. MoH Division of Clinical Practice, Kenya

19. National Aids Control Council (GoK). National Aids Control Council Strategic Plan. 2022. NACC, Kenya. Available from<https://nsdcc.go.ke/policies-strategies-and-guidelines/>

20. Ministry of health (GoK). Kenya Population Based HIV Impact Assessment (KENPHIA) Report. 2018

21. Katz IT, Maughan-Brown B. Improved life expectancy of people living with HIV: who is left behind? *Lancet HIV*. Aug 2017;4(8):e324-e326. doi:10.1016/s2352-3018(17)30086-3

22. UNAIDS. UNAIDS Data. April 25, 2022. Accessed April 25, 2022.<https://www.unaids.org/sites/default/files/media_asset/JC3032_AIDS_Data_book_2021_En.pdf>

23. Boulle A, Schomaker M, May MT, et al. Mortality in patients with HIV-1 infection starting antiretroviral therapy in South Africa, Europe, or North America: a collaborative analysis of prospective studies. *PLoS Med*. Sep 2014;11(9):e1001718. doi:10.1371/journal.pmed.1001718

24. Kenya National AIDS Control Council. Kenya HIV Estimates Report 2018. November 09, 2019. Accessed July 08, 2020.<https://nacc.or.ke/wp-content/uploads/2018/11/HIV-estimates-report-Kenya-20182.pdf>

25. Collins PY, Velloza J, Concepcion T, et al. Intervening for HIV prevention and mental health: a review of global literature. *J Int AIDS Soc*. Jun 2021;24 Suppl 2(Suppl 2):e25710. doi:10.1002/jia2.25710

26. Blakemore SJ. Adolescence and mental health. *Lancet*. May 18 2019;393(10185):2030-2031. doi:10.1016/s0140-6736(19)31013-x

27. Orth Z, van Wyk B. Measuring mental wellness among adolescents living with a physical chronic condition: a systematic review of the mental health and mental well-being instruments. *BMC Psychol*. Nov 8 2021;9(1):176. doi:10.1186/s40359-021-00680-w

28. Barker MM, Beresford B, Bland M, Fraser LK. Prevalence and Incidence of Anxiety and Depression Among Children, Adolescents, and Young Adults With Life-Limiting Conditions: A Systematic Review and Meta-analysis. *JAMA Pediatr*. Sep 1 2019;173(9):835-844. doi:10.1001/jamapediatrics.2019.1712

29. Butler A, Van Lieshout RJ, Lipman EL, et al. Mental disorder in children with physical conditions: a pilot study. *BMJ Open*. Jan 3 2018;8(1):e019011. doi:10.1136/bmjopen-2017-019011

30. Thabrew H, Stasiak K, Hetrick SE, et al. Psychological therapies for anxiety and depression in children and adolescents with long-term physical conditions. *Cochrane Database Syst Rev*. Dec 22 2018;12(12):Cd012488. doi:10.1002/14651858.CD012488.pub2

31. Collins PY, Holman AR, Freeman MC, Patel V. What is the relevance of mental health to HIV/AIDS care and treatment programs in developing countries? A systematic review. *Aids*. 2006:1571-82. vol. 12.

32. Dessauvagie AS, Jörns-Presentati A, Napp AK, et al. The prevalence of mental health problems in sub-Saharan adolescents living with HIV: a systematic review. *Glob Ment Health (Camb)*. 2020;7:e29. doi:10.1017/gmh.2020.18

33. Jörns-Presentati A, Napp AK, Dessauvagie AS, et al. The prevalence of mental health problems in sub-Saharan adolescents: A systematic review. *PLoS One*. 2021;16(5):e0251689. doi:10.1371/journal.pone.0251689

34. Olashore AA, Paruk S, Akanni OO, Tomita A, Chiliza B. Psychiatric Disorders in Adolescents Living with HIV and Association with Antiretroviral Therapy Adherence in Sub-Saharan Africa: A Systematic Review and Meta-analysis. *AIDS Behav*. Jun 2021;25(6):1711-1728. doi:10.1007/s10461-020-03100-z

35. Too EK, Abubakar A, Nasambu C, et al. Prevalence and factors associated with common mental disorders in young people living with HIV in sub-Saharan Africa: a systematic review. *J Int AIDS Soc*. Jun 2021;24 Suppl 2(Suppl 2):e25705. doi:10.1002/jia2.25705

36. Vreeman RC, McCoy BM, Lee S. Mental health challenges among adolescents living with HIV. *J Int AIDS Soc*. May 16 2017;20(Suppl 3):21497. doi:10.7448/ias.20.4.21497

37. Mellins CA, Malee KM. Understanding the mental health of youth living with perinatal HIV infection: lessons learned and current challenges. *J Int AIDS Soc*. Jun 18 2013;16(1):18593. doi:10.7448/ias.16.1.1859318593

38. Evangeli M. Mental health and substance use in HIV-infected adolescents. *Curr Opin HIV AIDS*. May 2018;13(3):204-211. doi:10.1097/coh.0000000000000451

39. Gaitho D, Kumar M, Wamalwa D, Wambua GN, Nduati R. Understanding mental health difficulties and associated psychosocial outcomes in adolescents in the HIV clinic at Kenyatta National Hospital, Kenya. *Annals of general psychiatry*. 2018;17(1):29.

40. Kamau JW, Kuria W, Mathai M, Atwoli L, Kangethe R. Psychiatric morbidity among HIV-infected children and adolescents in a resource-poor Kenyan urban community. *AIDS Care*. 2012;24(7):836-42. doi:10.1080/09540121.2011.644234

41. Molinaro M, Mwanza-Kabaghe S, Mweemba M, et al. Evaluating the Relationship Between Depression and HIV-associated Cognitive Impairment Among Children and Adolescents in Zambia (S7. 001). AAN Enterprises; 2019.

42. Lwidiko A, Kibusi SM, Nyundo A, Mpondo BCT. Association between HIV status and depressive symptoms among children and adolescents in the Southern Highlands Zone, Tanzania: A case-control study. *PLoS One*. 2018;13(2):e0193145. doi:10.1371/journal.pone.0193145

43. Bankole KO, Bakare MO, Edet BE, et al. Psychological complications associated with HIV/AIDS infection among children in South-South Nigeria, sub-Saharan Africa. *Cogent Medicine*. 2017;4(1):1372869.

44. Buckley J, Otwombe K, Joyce C, et al. Mental Health of Adolescents in the Era of Antiretroviral Therapy: Is There a Difference Between HIV-Infected and Uninfected Youth in South Africa? *J Adolesc Health*. Jul 2020;67(1):76-83. doi:10.1016/j.jadohealth.2020.01.010

45. Abubakar A, Van de Vijver FJR, Hassan AS, et al. Cumulative Psychosocial Risk is a Salient Predictor of Depressive Symptoms among Vertically HIV-Infected and HIV-Affected Adolescents at the Kenyan Coast. *Ann Glob Health*. Sep-Dec 2017;83(5-6):743-752. doi:10.1016/j.aogh.2017.10.024

46. Woollett N, Cluver L, Bandeira M, Brahmbhatt H. Identifying risks for mental health problems in HIV positive adolescents accessing HIV treatment in Johannesburg. *Journal of Child & Adolescent Mental Health*. 2017;29(1):11-26.

47. Mutumba M, Bauermeister JA, Harper GW, et al. Psychological distress among Ugandan adolescents living with HIV: Examining stressors and the buffering role of general and religious coping strategies. *Glob Public Health*. Dec 2017;12(12):1479-1491. doi:10.1080/17441692.2016.1170871

48. Boyes ME, Cluver LD, Meinck F, Casale M, Newnham E. Mental health in South African adolescents living with HIV: correlates of internalising and externalising symptoms. *AIDS Care*. Jan 2019;31(1):95-104. doi:10.1080/09540121.2018.1524121

49. Kim MH, Mazenga AC, Yu X, et al. Factors associated with depression among adolescents living with HIV in Malawi. *BMC psychiatry*. 2015;15(1):264.

50. Kemigisha E, Zanoni B, Bruce K, et al. Prevalence of depressive symptoms and associated factors among adolescents living with HIV/AIDS in South Western Uganda. *AIDS Care*. Oct 2019;31(10):1297-1303. doi:10.1080/09540121.2019.1566511

51. Abramowitz S, Koenig LJ, Chandwani S, et al. Characterizing social support: global and specific social support experiences of HIV-infected youth. *AIDS Patient Care STDS*. May 2009;23(5):323-30. doi:10.1089/apc.2008.0194

52. Mak WWS, Mo PKH, Ma GYK, Lam MYY. Meta-analysis and systematic review of studies on the effectiveness of HIV stigma reduction programs. *Soc Sci Med*. Sep 2017;188:30-40. doi:10.1016/j.socscimed.2017.06.045

53. Andersson GZ, Reinius M, Eriksson LE, et al. Stigma reduction interventions in people living with HIV to improve health-related quality of life. *Lancet HIV*. Feb 2020;7(2):e129-e140. doi:10.1016/s2352-3018(19)30343-1

54. DeAtley T, Harrison A, Mtukushe B, et al. Conditional Economic Incentives for HIV Treatment Adherence: Aligning Adolescent Developmental Hallmarks with Behavioral Economic Theory to Improve HIV Treatment Adherence. *AIDS Patient Care STDS*. Jul 2022;36(7):272-277. doi:10.1089/apc.2022.0060

55. Mutumba M, Musiime V, Lepkwoski JM, et al. Examining the relationship between psychological distress and adherence to anti-retroviral therapy among Ugandan adolescents living with HIV. *AIDS Care*. Jul 2016;28(7):807-15. doi:10.1080/09540121.2015.1131966

56. Sudfeld CR, Kaaya S, Gunaratna NS, et al. Depression at antiretroviral therapy initiation and clinical outcomes among a cohort of Tanzanian women living with HIV. *Aids*. Jan 14 2017;31(2):263-271. doi:10.1097/qad.0000000000001323

57. Smith Fawzi MC, Ng L, Kanyanganzi F, et al. Mental Health and Antiretroviral Adherence Among Youth Living With HIV in Rwanda. *Pediatrics*. Oct 2016;138(4)doi:10.1542/peds.2015-3235

58. Okawa S, Mwanza Kabaghe S, Mwiya M, et al. Psychological well-being and adherence to antiretroviral therapy among adolescents living with HIV in Zambia. *AIDS Care*. May 2018;30(5):634-642. doi:10.1080/09540121.2018.1425364

59. Dow DE, Turner EL, Shayo AM, Mmbaga B, Cunningham CK, O'Donnell K. Evaluating mental health difficulties and associated outcomes among HIV-positive adolescents in Tanzania. *AIDS Care*. Jul 2016;28(7):825-33. doi:10.1080/09540121.2016.1139043

60. Bucek A, Leu CS, Benson S, et al. Psychiatric Disorders, Antiretroviral Medication Adherence and Viremia in a Cohort of Perinatally HIV-Infected Adolescents and Young Adults. *Pediatr Infect Dis J*. Jul 2018;37(7):673-677. doi:10.1097/inf.0000000000001866

61. Ekat MH, Yotebieng M, Leroy V, et al. Association between depressive symptoms and adherence among adolescents living with HIV in the Republic of Congo: A cross sectional study. *Medicine (Baltimore)*. Aug 28 2020;99(35):e21606. doi:10.1097/md.0000000000021606

62. Jin M, An Q, Wang L. Chronic conditions in adolescents. *Exp Ther Med*. Jul 2017;14(1):478-482. doi:10.3892/etm.2017.4526

63. Bucek A, Mellins C, Abrams E. Helping youth with perinatal HIV thrive: Lessons learned from the first generation of youth living with perinatal HIV infection. *American Psychological Association Psychology and AIDS Exchange Newsletter*. 2019;

64. Lyambai K. *Mental health problems experienced by HIV postive adoloscents: a case of Choma district, Zambia*. The University of Zambia; 2017.

65. Sin NL, DiMatteo MR. Depression treatment enhances adherence to antiretroviral therapy: a meta-analysis. *Ann Behav Med*. Jun 2014;47(3):259-69. doi:10.1007/s12160-013-9559-6

66. Simoni JM, Wiebe JS, Sauceda JA, et al. A preliminary RCT of CBT-AD for adherence and depression among HIV-positive Latinos on the U.S.-Mexico border: the Nuevo Día study. *AIDS Behav*. Oct 2013;17(8):2816-29. doi:10.1007/s10461-013-0538-5

67. Safren SA, Bedoya CA, O'Cleirigh C, et al. Cognitive behavioural therapy for adherence and depression in patients with HIV: a three-arm randomised controlled trial. *Lancet HIV*. Nov 2016;3(11):e529-e538. doi:10.1016/s2352-3018(16)30053-4

68. Brown LK, Kennard BD, Emslie GJ, et al. Effective Treatment of Depressive Disorders in Medical Clinics for Adolescents and Young Adults Living With HIV: A Controlled Trial. *J Acquir Immune Defic Syndr*. Jan 1 2016;71(1):38-46. doi:10.1097/qai.0000000000000803

69. Chuah FLH, Haldane VE, Cervero-Liceras F, et al. Interventions and approaches to integrating HIV and mental health services: a systematic review. *Health Policy Plan*. Nov 1 2017;32(suppl_4):iv27-iv47. doi:10.1093/heapol/czw169

70. Hammett WH, Muanido A, Cumbe VFJ, et al. Demonstration project of a lay counselor delivered trans-diagnostic mental health intervention for newly diagnosed HIV patients in Mozambique. *AIDS Care*. Mar 29 2022:1-6. doi:10.1080/09540121.2022.2039356

71. Remien RH, Stirratt MJ, Nguyen N, Robbins RN, Pala AN, Mellins CA. Mental health and HIV/AIDS: the need for an integrated response. *Aids*. 2019:1411-1420. vol. 9.

72. Chi P, Zhao S, Zhang C, et al. Effects of psychosocial interventions on children affected by parental HIV/AIDS: a meta-analysis on depression and anxiety. *BMC Public Health*. Nov 27 2019;19(1):1572. doi:10.1186/s12889-019-7806-x

73. Chibanda D, Weiss HA, Verhey R, et al. Effect of a Primary Care-Based Psychological Intervention on Symptoms of Common Mental Disorders in Zimbabwe: A Randomized Clinical Trial. *Jama*. Dec 27 2016;316(24):2618-2626. doi:10.1001/jama.2016.19102

74. Dorsey S, Lucid L, Martin P, et al. Effectiveness of Task-Shifted Trauma-Focused Cognitive Behavioral Therapy for Children Who Experienced Parental Death and Posttraumatic Stress in Kenya and Tanzania: A Randomized Clinical Trial. *JAMA Psychiatry*. May 1 2020;77(5):464-473. doi:10.1001/jamapsychiatry.2019.4475

75. Nakimuli-Mpungu E, Musisi S, Wamala K, et al. Effectiveness and cost-effectiveness of group support psychotherapy delivered by trained lay health workers for depression treatment among people with HIV in Uganda: a cluster-randomised trial. *Lancet Glob Health*. Mar 2020;8(3):e387-e398. doi:10.1016/s2214-109x(19)30548-0

76. Fuller SM, Koester KA, Erguera XA, et al. The collaborative care model for HIV and depression: Patient perspectives and experiences from a safety-net clinic in the United States. *SAGE Open Med*. 2019;7:2050312119842249. doi:10.1177/2050312119842249

77. Unützer J, Carlo AD, Collins PY. Leveraging collaborative care to improve access to mental health care on a global scale. *World Psychiatry*. Feb 2020;19(1):36-37. doi:10.1002/wps.20696

78. Bhana A, Kreniske P, Pather A, Abas MA, Mellins CA. Interventions to address the mental health of adolescents and young adults living with or affected by HIV: state of the evidence. *J Int AIDS Soc*. Jun 2021;24 Suppl 2(Suppl 2):e25713. doi:10.1002/jia2.25713

79. Bhana A, Abas MA, Kelly J, van Pinxteren M, Mudekunye LA, Pantelic M. Mental health interventions for adolescents living with HIV or affected by HIV in low- and middle-income countries: systematic review. *BJPsych Open*. Sep 4 2020;6(5):e104. doi:10.1192/bjo.2020.67

80. UNAIDS. Prevailing Against Pandemics By Putting People at the Centre: World AIDS Day Report, 2020. August 02, 2022.<https://www.unaids.org/sites/default/files/media_asset/prevailing-against-pandemics_en.pdf>

81. World Health Organization. Consolidated guidelines on HIV prevention, testing, treatment, service delivery and monitoring: recommendations for a public health approach. September 10, 2021. Accessed September 10, 2021.<https://www.who.int/publications/i/item/9789240031593>

82. Ministry of Health Kenya. Guideline On Use of Antiretroviral Drugs for Treating and Preventing HIV in Kenya. October 17, 2019. Accessed October 17 2019,<http://cquin.icap.columbia.edu/wp-content/uploads/2017/04/ICAP_CQUIN_Kenya-ARV-Guidelines-2018-Final_20thAug2018.pdf>

83. Ministry of Health. Kenya Mental Health Policy 2015-2030. MOH. January 18, 2022. Accessed January 18, 2022.<https://publications.universalhealth2030.org/uploads/Kenya-Mental-Health-Policy.pdf>

84. World Health Organization. Making health services adolescent friendly: developing national quality standards for adolescent friendly health services. 2012;

85. Ministry of Health. National Guidelines for Provision of Adolescent and Youth Friendly Services in Kenya. July 29, 2020. Accessed October 24, 2019.<https://faces.ucsf.edu/sites/g/files/tkssra4711/f/YouthGuidelines2016.pdf>

86. Njuguna I, Beima-Sofie K, Mburu C, et al. What happens at adolescent and young adult HIV clinics? A national survey of models of care, transition and disclosure practices in Kenya. *Trop Med Int Health*. May 2020;25(5):558-565. doi:10.1111/tmi.13374

87. Mendenhall E, De Silva MJ, Hanlon C, et al. Acceptability and feasibility of using non-specialist health workers to deliver mental health care: stakeholder perceptions from the PRIME district sites in Ethiopia, India, Nepal, South Africa, and Uganda. *Social science & medicine*. 2014;118:33-42.

88. Burmen B, Owuor N, Mitei P. An assessment of staffing needs at a HIV clinic in a Homa Bay using the WHO workload indicators of staffing need WISN, 2011. *Hum Resour Health*. Jan 26 2017;15(1):9. doi:10.1186/s12960-017-0186-3

89. Mugo C, Wilson K, Wagner AD, et al. Pilot evaluation of a standardized patient actor training intervention to improve HIV care for adolescents and young adults in Kenya. *AIDS Care*. Oct 2019;31(10):1250-1254. doi:10.1080/09540121.2019.1587361

90. Catherine Barker, Aaron Mulaki, Daniel Mwai, Arin Dutta, Health Policy Project, Group F. Devolution of Healthcare in Kenya: Assessing County Health System Readiness in Kenya: A review of Selected Health Inputs. Accessed September 16, 2018.<https://www.healthpolicyproject.com/pubs/479_KenyaPETSCountyReadinessFINAL.pdf>

91. Ministry of Health. Mental Health and Wellbeing: A report by the taskforce on mental health in Kenya. MOH. January 18, 2022. Accessed January 18, 2022.<https://www.pdokenya.org/uploads/8/0/2/8/80287644/taskforce_on_mental_health_report__web___1_.pdf>

92. Barasa E, Nguhiu P, McIntyre D. Measuring progress towards sustainable development goal 3.8 on universal health coverage in Kenya. *BMJ global health*. 2018;3(3):e000904.

93. Sirengo M, Muthoni L, Kellogg TA, et al. Mother-to-child transmission of HIV in Kenya: results from a nationally representative study. *J Acquir Immune Defic Syndr*. May 1 2014;66 Suppl 1:S66-74. doi:10.1097/qai.0000000000000115

94. Government of Kenya. Guidelines for prevention of mother to child transmission of HIV/AIDS in Kenya, 4th edition, 2012. July 21, 2019. Accessed July, 21, 2019.<http://guidelines.health.go.ke:8000/media/Guidelines_for_PMTCT_of_HIVAIDS_in_Kenya-1.pdf>

95. Ministry of Health. The Kenya HIV Testing Guidelines. Ministry of Health. Accessed July, 21, 2019.<https://aidsfree.usaid.gov/sites/default/files/hts_policy_kenya_2015.pdf>

96. Guidelines for antiretroviral use for HIV treatment and prevention in Kenya (2018).

97. Lahuerta M, Syowai M, Vakil S, et al. Monitoring the transition to new antiretroviral treatment regimens through an enhanced data system in Kenya. *PLoS One*. 2020;15(4):e0232104. doi:10.1371/journal.pone.0232104

98. Mburu M, Guze MA, Ong'wen P, et al. Evaluating the effectiveness of the HIV adolescent package of care (APOC) training on viral load suppression in Kenya. *Public Health*. Aug 2019;173:146-149. doi:10.1016/j.puhe.2019.05.026

99. Wilson KS, Mugo C, Moraa H, et al. Health provider training is associated with improved engagement in HIV care among adolescents and young adults in Kenya. *Aids*. Jul 15 2019;33(9):1501-1510. doi:10.1097/qad.0000000000002217

100. Rech D, Bertrand JT, Thomas N, et al. Surgical efficiencies and quality in the performance of voluntary medical male circumcision (VMMC) procedures in Kenya, South Africa, Tanzania, and Zimbabwe. *PLoS One*. 2014;9(5):e84271. doi:10.1371/journal.pone.0084271

101. Mark D, Hrapcak S, Ameyan W, et al. Peer Support for Adolescents and Young People Living with HIV in sub-Saharan Africa: Emerging Insights and a Methodological Agenda. *Curr HIV/AIDS Rep*. Dec 2019;16(6):467-474. doi:10.1007/s11904-019-00470-5

102. Marangu E, Sands N, Rolley J, Ndetei D, Mansouri F. Mental healthcare in Kenya: exploring optimal conditions for capacity building. *Afr J Prim Health Care Fam Med*. Oct 10 2014;6(1):E1-5. doi:10.4102/phcfm.v6i1.682

103. Mutiso V, Pike K, Musyimi C, et al. Feasibility and effectiveness of nurses and clinical officers in implementing the WHO mhGAP intervention guide: Pilot study in Makueni County, Kenya. *General hospital psychiatry*. 2019;59:20-29.

104. Apondi E, Wachira J, Ayikukwei R, et al. Barriers to ART adherence among school students living with HIV in Kenya. *Afr J AIDS Res*. Oct 2021;20(3):232-237. doi:10.2989/16085906.2021.1979606

105. Kose J, Lenz C, Akuno J, et al. Supporting adolescents living with HIV within boarding schools in Kenya. *PLoS One*. 2021;16(12):e0260278. doi:10.1371/journal.pone.0260278

106. Ejeby K, Savitskij R, Ost LG, et al. Randomized controlled trial of transdiagnostic group treatments for primary care patients with common mental disorders. *Fam Pract*. Jun 2014;31(3):273-80. doi:10.1093/fampra/cmu006

107. Kessler RC, Chiu WT, Demler O, Merikangas KR, Walters EE. Prevalence, severity, and comorbidity of 12-month DSM-IV disorders in the National Comorbidity Survey Replication. *Arch Gen Psychiatry*. Jun 2005;62(6):617-27. doi:10.1001/archpsyc.62.6.617

108. Dalgleish T, Black M, Johnston D, Bevan A. Transdiagnostic approaches to mental health problems: Current status and future directions. *J Consult Clin Psychol*. Mar 2020;88(3):179-195. doi:10.1037/ccp0000482

109. Garber J, Brunwasser SM, Zerr AA, Schwartz KT, Sova K, Weersing VR. Treatment and Prevention of Depression and Anxiety in Youth: Test of Cross-Over Effects. *Depress Anxiety*. Oct 2016;33(10):939-959. doi:10.1002/da.22519

110. Murray LK, Kane JC, Glass N, et al. Effectiveness of the Common Elements Treatment Approach (CETA) in reducing intimate partner violence and hazardous alcohol use in Zambia (VATU): A randomized controlled trial. *PLoS Med*. Apr 2020;17(4):e1003056. doi:10.1371/journal.pmed.1003056

111. Murray LK, Haroz E, Dorsey S, Kane J, Bolton PA, Pullmann MD. Understanding mechanisms of change: An unpacking study of the evidence-based common-elements treatment approach (CETA) in low and middle income countries. *Behav Res Ther*. Jul 2020;130:103430. doi:10.1016/j.brat.2019.103430

112. Fabian KE, Muanido A, Cumbe VFJ, et al. Integrating a Transdiagnostic Psychological Intervention Into Routine HIV Care: A Mixed-Methods Evaluation of the Common Elements Treatment Approach in Mozambique. *J Acquir Immune Defic Syndr*. Mar 1 2022;89(3):274-281. doi:10.1097/qai.0000000000002863

113. Barlow DH, Farchione TJ, Bullis JR, et al. The Unified Protocol for Transdiagnostic Treatment of Emotional Disorders Compared With Diagnosis-Specific Protocols for Anxiety Disorders: A Randomized Clinical Trial. *JAMA Psychiatry*. Sep 1 2017;74(9):875-884. doi:10.1001/jamapsychiatry.2017.2164

114. Lyon AR, Bruns EJ, Ludwig K, et al. The Brief Intervention for School Clinicians (BRISC): A mixed-methods evaluation of feasibility, acceptability, and contextual appropriateness. *School Ment Health*. Dec 1 2015;7(4):273-286. doi:10.1007/s12310-015-9153-0

115. Simms V, Weiss HA, Chinoda S, et al. Peer-led counselling with problem discussion therapy for adolescents living with HIV in Zimbabwe: A cluster-randomised trial. *PLoS Med*. Jan 2022;19(1):e1003887. doi:10.1371/journal.pmed.1003887

116. Raviola G, Naslund JA, Smith SL, Patel V. Innovative Models in Mental Health Delivery Systems: Task Sharing Care with Non-specialist Providers to Close the Mental Health Treatment Gap. *Curr Psychiatry Rep*. Apr 30 2019;21(6):44. doi:10.1007/s11920-019-1028-x

117. Kohrt BA, Ramaiya MK, Rai S, Bhardwaj A, Jordans MJD. Development of a scoring system for non-specialist ratings of clinical competence in global mental health: a qualitative process evaluation of the Enhancing Assessment of Common Therapeutic Factors (ENACT) scale. *Glob Ment Health (Camb)*. 2015;2doi:10.1017/gmh.2015.21

118. Kohrt BA, Jordans MJ, Rai S, et al. Therapist competence in global mental health: Development of the ENhancing Assessment of Common Therapeutic factors (ENACT) rating scale. *Behav Res Ther*. Jun 2015;69:11-21. doi:10.1016/j.brat.2015.03.009

119. Kohrt BA, Schafer A, Willhoite A, et al. Ensuring Quality in Psychological Support (WHO EQUIP): developing a competent global workforce. *World Psychiatry*. 2020:115-116. vol. 1.

120. Jordans MJD, Steen F, Koppenol-Gonzalez GV, et al. Evaluation of competency-driven training for facilitators delivering a psychological intervention for children in Lebanon: a proof-of-concept study. *Epidemiol Psychiatr Sci*. Jul 11 2022;31:e48. doi:10.1017/s2045796022000348

121. Wainberg ML, McKinnon K, Mattos PE, et al. A model for adapting evidence-based behavioral interventions to a new culture: HIV prevention for psychiatric patients in Rio de Janeiro, Brazil. *AIDS Behav*. Nov 2007;11(6):872-83. doi:10.1007/s10461-006-9181-8

122. OPTICC Center. Optimizing Implementation In Cancer Control (OPTICC) Methods. February 24, 2022. Accessed February 24, 2022.<https://www.opticc.org/our-methods/>

123. Murray LK, Dorsey S, Bolton P, et al. Building capacity in mental health interventions in low resource countries: an apprenticeship model for training local providers. *Int J Ment Health Syst*. Nov 18 2011;5(1):30. doi:10.1186/1752-4458-5-30

124. Njuguna IN, Beima-Sofie K, Mburu CW, et al. Adolescent transition to adult care for HIV-infected adolescents in Kenya (ATTACH): study protocol for a hybrid effectiveness-implementation cluster randomised trial. *BMJ Open*. Dec 2 2020;10(12):e039972. doi:10.1136/bmjopen-2020-039972

125. Wagner AD, Mugo C, Bluemer-Miroite S, et al. Continuous quality improvement intervention for adolescent and young adult HIV testing services in Kenya improves HIV knowledge. *Aids*. Jul 1 2017;31 Suppl 3:S243-s252. doi:10.1097/qad.0000000000001531

126. Weiner BJ, Lewis CC, Stanick C, et al. Psychometric assessment of three newly developed implementation outcome measures. *Implement Sci*. Aug 29 2017;12(1):108. doi:10.1186/s13012-017-0635-3
